# Mono- and Di-Quaternized 4,4′-Bipyridine Derivatives as Key Building Blocks for Medium- and Environment-Responsive Compounds and Materials

**DOI:** 10.3390/molecules25010001

**Published:** 2019-12-18

**Authors:** Raffaello Papadakis

**Affiliations:** 1 School of Chemical Engineering, National Technical University of Athens (NTUA), Laboratory of Organic Chemistry, 15780 Athens, Greece; rafpapadakis@gmail.com; Tel.: +46-728-368-595; 2 Department of Chemistry—Ångström Laboratory, Uppsala University, 751 20 Uppsala, Sweden

**Keywords:** viologens, monoquats, solvatochromism, thermochromism, medium-responsive compounds, environment-responsive materials.

## Abstract

Mono- and di-quaternized 4,4′-bipyridine derivatives constitute a family of heterocyclic compounds, which in recent years have been employed in numerous applications. These applications correspond to various disciplines of research and technology. In their majority, two key features of these 4,4′-bipyridine-based derivatives are exploited: their redox activity and their electrochromic aptitude. Contemporary materials and compounds encompassing these skeletons as building blocks are often characterized as multifunctional, as their presence often gives rise to interesting phenomena, e.g., various types of chromism. This research trend is acknowledged, and, in this review article, recent examples of multifunctional chromic materials/compounds of this class are presented. Emphasis is placed on solvent-/medium- and environment-responsive 4,4′-bipyridine derivatives. Two important classes of 4,4′-bipyridine-based products with solvatochromic and/or environment-responsive character are reviewed: viologens (i.e., *N*,*N′*-disubstituted derivatives) and monoquats (i.e., monosubstituted 4,4′-bipyridine derivatives). The multifunctional nature of these derivatives is analyzed and structure–property relations are discussed in connection to the role of these derivatives in various novel applications.

## 1. Introduction

**Figure 1:**
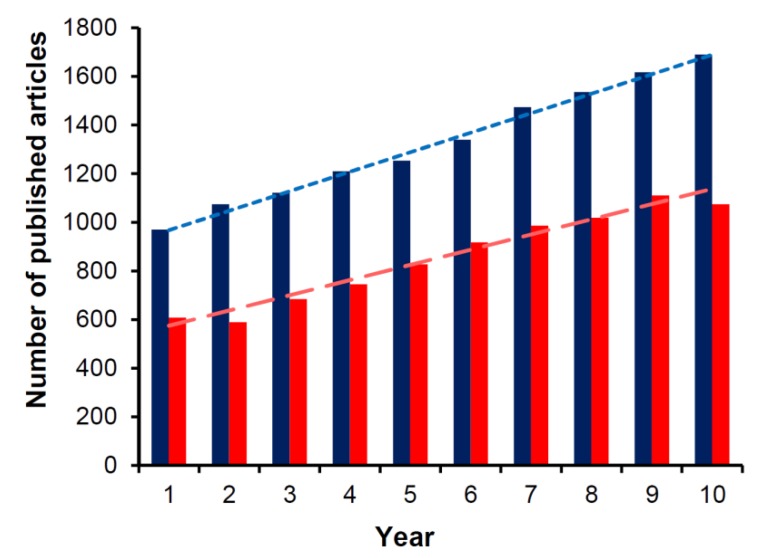
Plot depicting the linear increase of research papers involving the terms “viologen” (blue) and “solvatochromism” (red) from years 2009 (year 1) to year 2018 (year 10). Source: SCOPUS.

**Figure 2:**
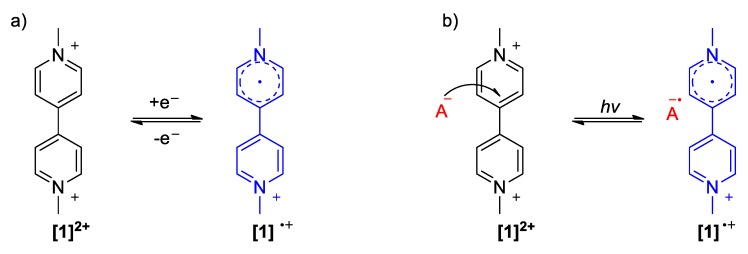
Schemes depicting (**a**) the electrochromic cycle (**1**) and (**b**) the photochromic cycle of paraquat (A, counter anion).

4,4′-Bipyridine is a heterocyclic compound, which is widely utilized as a building block in numerous materials and compounds [1]. In recent years, the derivatives of 4,4′-bipyridine have been employed in numerous high-tech applications including molecular motors [2], machines [3,4], and switches [5,6]. 4,4′-Bipyridine is highly abundant in supramolecular chemistry as well, where it mainly serves as an electro- and photo-sensitive building block as well as a bidentate coordinating ligand, very often appearing in covalent organic and metalorganic frameworks (COFs and MOFs, respectively) [7,8,9]. A key characteristic of the derivatives of this important building block is their multifunctionality [10,11]. The most prominent class of 4,4′-bipyridine is that of viologens, i.e., the *N*,*N*′-disubstituted 4,4′-bipyridines, also known as paraquats, due to the presence of two quaternary nitrogen atoms of the two pyridine rings lying at para-positions. Viologen-based materials often exhibit very interesting electronic, optical, electrochemical, and magnetic properties [1,9,10], which can be combined in novel multifunctional systems suitable for a wide range of applications. There is a constant increase of published research works involving viologens (see Figure 1) and this perhaps is associated with the need for new materials corresponding to a variety of hi-tech applications. The importance of viologens is reflected through the numerous review papers (many of them very recent) underlining the high versatility of this class of compounds [9,10,12,13,14,15]. Perhaps, the most reviewed subject regarding the viologens is their electrochromism [13,14,16]. Viologens are known to undergo one-electron reductions electrochemically (Figure 2) yielding colorful radicals i.e., cations that are stable in the absence of O_2_. Yet, their chromism can also be triggered through various stimuli (including light, heat, solvents, presence of various chemical species, pressure, etc.). All these possibilities shape the very versatile “chromic character” of viologens and generally of 4,4′-bipyridine derivatives. Even though this “chromic character” has only recently started gaining more attention and there is an increasing number of research articles emphasizing on the alternative uses of 4,4′-bipyridine derivatives, there are no reviews in this growing area up until now. Such an attempt is presented in this review paper with an emphasis on medium- and environment-responsive compounds and materials based on 4,4′-bipyridine. Primary focus is placed on viologen-involving solvatochromic systems as well as materials and compounds of this class whose chromic behavior is dependent on various environment stimuli.

## 2. Structure and Properties of Viologens

### 2.1. The Strong Electron-Withdrawing Character of Viologens

The strong electron-withdrawing character of viologens has been widely exploited in numerous molecular architectures and materials. Viologens can undergo reversible one-electron reductions forming radical cations (Figure 2a), which are vividly colored often blue or violet depending on the substituents of the quaternized N-atoms. The reduction potential of a viologen varies depending on its substituents with that of the simplest viologen (or simply paraquat) and is found to be −350 mV (versus SCE) [17]. The formation of the radical cations is obvious as the solutions of the diquaternized forms of viologens are in most of the cases colorless or light yellow. The aforementioned chemical change is narrowly associated with the electro- and photo-chromism of viologens [16,18]. These radical cation species are stable under unaerated conditions and inclusion of viologens in polymer matrices can often yield materials with high switching aptitude and longer life [18]. The described one-electron reduction can be triggered in various ways including the use of reducing agents, e.g., dithionite ions [19], photochemically [20] or using other chemical species that readily form charge transfer complexes with viologens, e.g., I^−^ [18], tetrathiafulvalene (TTF) [21], phenols [22,23], etc. In all these cases, the reducing species transfer one electron to the electron-deficient viologen skeleton in an inter- or intra-molecular fashion. Light irradiation can trigger charge transfer from the counter anion of a viologen (BPh_4_)^−^ [24], [Fe^II^(CN)_6_]^4−^ [25], etc. (Figure 2b) and the photochromism of viologens is attributed to this effect [18]. The stability of the PQ^+.^/A^−^ can vary depending on the nature of the anion A. A very important class of medium-responsive and environment-dependent chromic viologen-based systems corresponding to the aforementioned case is that of charge transfer complexes (CTCs), which are reviewed in Section 5.2. Moreover, the color of the viologen radical cations in solution is highly affected by solvent polarity as well as the nature of the N-substituents. All these effects can lead to multifunctional systems for the control of which different parameters can be exploited.

### 2.2. Dihedral Angle Importance in Optical and Electronic Applications

**Figure 3:**
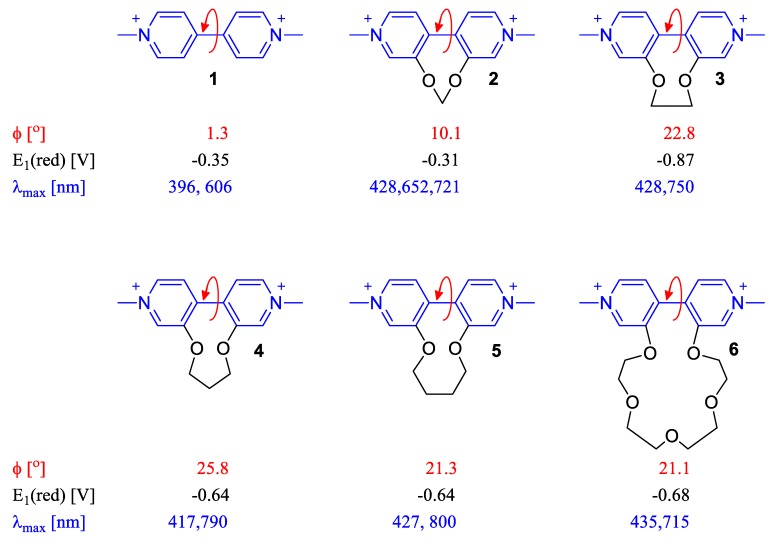
Paraquat (**1**) and viologen derivatives (**2**–**6**) synthesized and studied by Benniston et al. In red is the dihedral angle between the two pyridine rings of each viologen dication, in black is the reduction potential for the transformation of a viologen dication to the corresponding radical cation and in blue is the absorption maxima wavelengths of the corresponding radical cations [17].

The dihedral angle between the two pyridinium rings in a viologen demonstrates a very important role in its electronic, electrochemical, and optical properties as it can influence the “communication” of the two rings and affect the stabilization of the viologen radical cation formed upon reduction. This effect is very crucial for the chromic behavior of a viologen as it will be analyzed in various examples in the next sections. Benniston et al. prepared five viologens involving tethering chains attached at positions 3,3′ as shown in Figure 3 [17]. Depending on the length of the tethering chains, different dihedral angles were observed for each of these five viologens. It was observed that not only the electrochemical one-electron reduction potentials of these viologens were altered when compared to parent paraquat (**1**) but also the spectral characteristics (absorption maxima and molar extinction coefficients) of the formed radical cations. The situation becomes even more interesting when investigating the fluorescence of viologens (especially the ones of low molecular weight), a research topic which has been debated for a long time [1,26,27,28]. The prevailing belief has been that viologens are non-fluorescent [1]. The interpretation for the lack of emission is often related to the labile positions 2-, 2′- and 6-, 6′- (or ortho-positions), which can be susceptible to oxidation. Yet, it is known that conformational changes that a viologen can undergo (e.g., twisting around the bond of the two pyridinium rings) may compete with its emissive behavior. Enclosing viologen molecules in cavities, e.g., in zeolites [29] or including viologen units in bulky polymers [30] and clays [31] is therefore supposed to enhance the emissive character of viologens not only by blocking side-reactions at the ortho-positions but also by locking the desired emissive conformations (i.e., right dihedral angle). Such observations have been also made by Scaiano and coworkers [32]. Interestingly, the importance of the twisting angle of the 4,4′-bipyridine skeleton can also be high in various electronic applications. Bâldea et al. have demonstrated through quantum chemical calculations that the twisted molecular conformations of the 4,4′-bipyridine skeleton can readily influence its conductance [33]. Taken together, the dihedral angle between the pyridine rings in a viologen is an important parameter, which is taken into account when designing new products for applications requiring high responsiveness to external (environment) stimuli, e.g., electricity, light, heat, pressure, solvent polarity, etc.

## 3. Direct Medium- and Environment-Responsive Viologens

**Figure 4:**
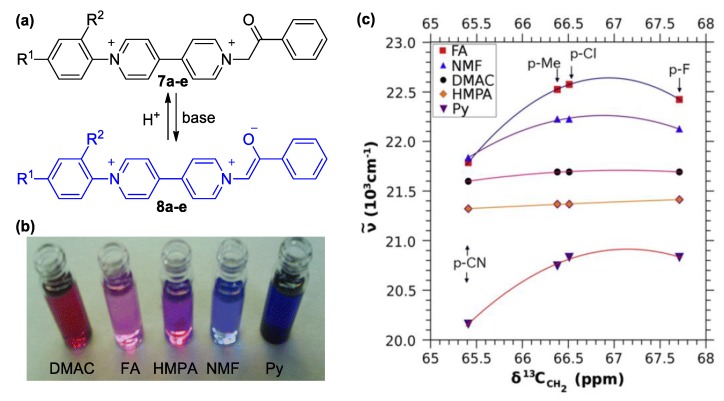
(**a**) The unsymmetric viologen PF_6_^−^ salts synthesized by Papadakis et al. [35,36] and the corresponding solvatochromic pyridinium enolate formed in basic solvents (**7a**: R^1^ = Me, R^2^ = H; **7b**: R^1^ = F, R^2^ = H; **7c**: R^1^= Cl, R^2^ = H; **7d**: R^1^= CN, R^2^ = H; **7e**: R^1^ = NO_2_, R^2^ = NO_2_;). (**b**) Photograph depicting the change in color observed when shifting to solvents of higher basicity (left to right). Author’s own photograph. (**c**) Plot indicating the correlation between the measured maximum charge transfer wavenumber of viologens **7**(**a**–**e**) and ^13^C-NMR chemical shift of the methylene groups of the same viologens. Reproduced with permission from [36]. Copyright Elsevier.

Even though there are various examples of viologen-based materials/compounds exhibiting a medium-responsive behavior, most of the examples involve viologens and another “molecular partner,” e.g., counter anions that are capable of transferring electron(s) to the dicationic viologen or fluorescent and chromogenic species that may exhibit chromic behavior themselves (e.g., solvatofluorochromism) [34]. In those cases, viologens demonstrate an important role by acting as strong electron acceptors as well as by efficiently stabilizing colored radical cations. In many cases, they also enhance the solvatochromic behavior of their chromic partner (molecules/other species). Such examples constitute a family of materials/compounds with a chromic behavior that is not purely viologen-mediated. For the sake of simplicity, such phenomena/systems will be referred to as indirect solvatochromic throughout this work. Yet, in this section, direct chromic viologen-mediated phenomena will be reviewed. Some years ago, Papadakis et al. reported on one of the first families of direct solvatochromic viologens [35,36]. These viologens were non-symmetric encompassing one aryl substituent on one *N*-atom and a phenacetyl group on the other *N*-atom (Figure 4, compounds **7a**–**7e**). The two strong electron-withdrawing groups neighboring to the methylene of the phenacetyl functionality (viologen quaternized *N*-atom and carbonyl group) in compounds **7a**–**7e** activate the methylene H-atoms and deprotonation is rendered possible in the presence of a Lewis base such as morpholine. The color of the isolated compounds **7a**–**7e** is typically beige to light yellow. Yet, when dissolved in solvents with significant HBA/Lewis-basicity such as hexamethyl phosphoramide (HMPA), dimethylacetamide (DMAC), dimethylsulfoxide (DMSO), *N*,*N*-dimethylformamide (DMF), *N*-methylformamide (NMF), or pyridine, the solutions of the aforementioned viologens become deeply colored and the color is highly dependent on the dipolarity and the basicity of the solvent (see Figure 4b). What makes these compounds interesting is that their colored solutions are stable for a long time even in the presence of O_2_. In fact, all UV–Vis measurements can be performed under aerated conditions. The color is attributed to the solvent-polarity-dependent charge transfer of the as-formed betaines, and the absorption maxima wavenumbers correlate fairly well with solvent polarity parameters expressing the basicity of solvents (Gutmann′s donor numbers: DN [37] and parameter β expressing HBA-basicity involved in the so-called Kamlet–Taft–Abboud solvatochromic equation [38]). The same research group also studied the correlations of the visible-solvatochromic shifts and the ^13^C-NMR chemical shifts attributed to the *N*-vicinal methylene group (Figure 4c). They concluded that these correlations indicate a narrow connection between the solvatochromic behavior and the structure of the betaines (compounds **8a**–**8e**).

**Figure 5:**
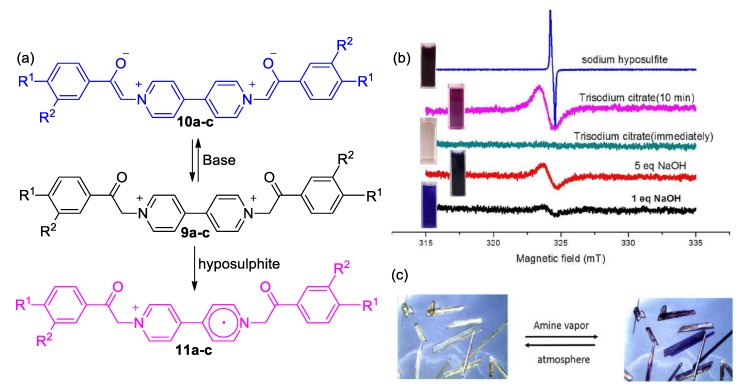
(**a**) The viologen cations by Zhu and coworkers (middle) and their reaction with a base to afford a solvatochromic enolate or hyposulphite to yield a radical cation (**9**–**11a**: R_1_ = R_2_ = H; **9**–**11b**: R_1_ = R_2_ = Cl; **9**–**11c**: R_1_ = R_2_ = OH). (**b**) The effect of base (NaOH_aq_), trisodium citrate, and sodium hyposulphite on the EPR spectra of viologen **9a**. (**c**) Color change of crystals of **9a** upon exposure to amine vapors. Reproduced with permission from [39]. Copyright American Chemical Society.

In a similar fashion, Zhu and coworkers reported, some years later, on a family of diphenacetyl viologen with high sensitivity to Lewis bases and solvents with significant basicity (Figure 5) [39]. The reported compounds (**9a**–**c**) are multifunctional as they can undergo reversible electrochromic and photochromic cycles. Importantly, these viologen-based sensors can operate both in the solid state and in solution phase in the absence or presence of air. As a further application, the same research group investigated the vapochromic aptitude of crystals of these viologens and found that after exposure of the crystals in vapors of amines, they became deep violet (Figure 5c). This outstanding chromic behavior is attributed to the formation of the corresponding betaines upon deprotonation of the *N*-vicinal methylene groups. Zhu and coworkers also performed EPR studies indicating charge transfer from the enolate to the electron-withdrawing part of these compounds, i.e., the 4,4′-bipyridine skeleton (Figure 5a). The intensity of the EPR signal attributed to the formation of radical cations was found to be highly dependent on basicity (Figure 5b). Even more intense EPR signals appear in the presence of hyposulphite, which can act as a reducing agent and promote the formation of viologen radical cations [39]. This finding indicated that the same viologen can undergo electrochromism as well as solvatochromism and vapochromism as a result of responses to different stimuli.

**Figure 6:**
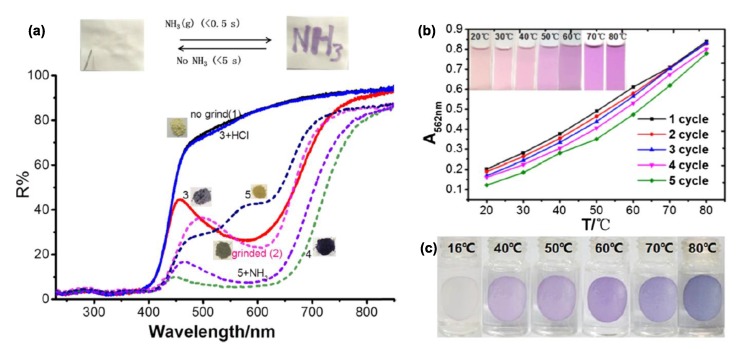
(**a**) (**Top**) 1.0 × 10^−4^ M aqueous solution of viologen **12** “written” on a piece of filter paper giving instant response to ammonia vapors; (**bottom**) the reflectance spectra of **12** prior to and after grinding or exposure to ammonia fumes. Thermochromic responses of **12** (**b**) in solution and (**c**) solid state from [40]. Copyright American Chemical Society.

Furthermore, the same research group later on reported on the special case of an ortho-phenolacetyl-substituted viologen (**12**) [40]. The latter has a high aptitude to coordinate various metal ions such as Fe(III), Cu(II), and Zn(II), and at the same time, it behaves as a strongly electron-deficient compound [40]. These features render this compound a perfect candidate for visual sensing of solvents, bases, and temperature in organic solvents as it exhibits solvato-, baso-, and thermo-chromism, respectively. Interestingly, this multifunctional compound responds to environment changes, e.g., temperature or NH_3_ vapors in its solid state but operates also in solution (Figure 6a,b). As also observed, in case of compounds **7a**–**7e** and **9a**–**9c**, the color changes are attributable to the enolate/pyridinium system and/or radical cation structure.

**Figure 7:**
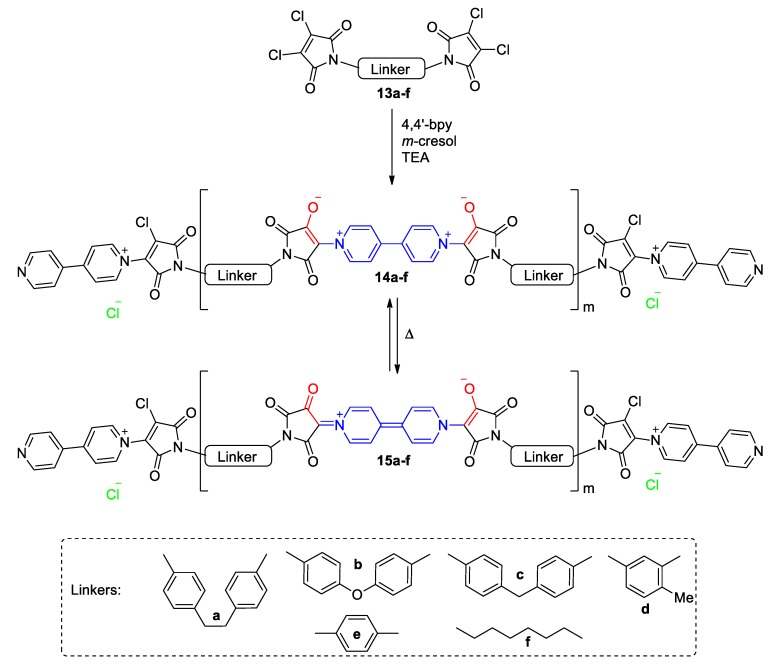
Synthetic route and thermochromic cycle of viologen polymers **14a**–**14f** by Gaina et al. [42].

The common structural characteristic of the chromic viologens **7a**–**7e** and **9a**–**9c** (Figure 4, Figure 5 and Figure 6) is the existence of an enolate in conjugation with the 4,4′-bipyridine scaffold. Pyridinium enolates have similarities in their solvatochromism with pyridinium phenolates (perhaps the most prominent example of this class of solvatochromic compounds is Reichardt’s dye [34,41]). However, 4,4′-Bipyridine is scarcely utilized as a scaffold for new dyes of that type. In an earlier publication by Gaina et al., a group of polymers with repeated viologen-enolate units were synthesized and isolated (Figure 7) [42]. In order to gain extended π-conjugation, Gaina et al. reacted 4,4-bipyridine with various tetrachloro-bismaleimides (Figure 7) to obtain polymers (**14a**–**14f**). Linking two of the aforementioned tetrachloro-bismaleimides requires a 4,4-bipyridine unit. The neighboring C-atom to the viologen-substituted carbon is sp^2^-hybridized (vinyl), is chlorosubstituted, yet under basic conditions yields an enolate. The as-formed enolate (highlighted red in Figure 7) is π-conjugated to the viologen unit (highlighted blue). The polymers typically exhibit a strong vis-band at 430 nm and a shoulder at 510 nm. In a similar fashion as in products **7a**–**7e** and **9a**–**9c,** the push–pull systems formed are expected to generate chromic effects. The authors hint that the products are thermochromic as heating can affect the CT and potentially the color of the compounds. They also identified a photo- and halo-chromic behavior. Specifically, the color of an alkaline solution of each of the polymers is blue-green; however, the presence of a strong base leads to decomposition of the polymers [42]. The compounds are very interesting candidates for chromic applications; however, they exhibit low solubility in organic solvents, which limits their in-solution applications.

**Figure 8:**
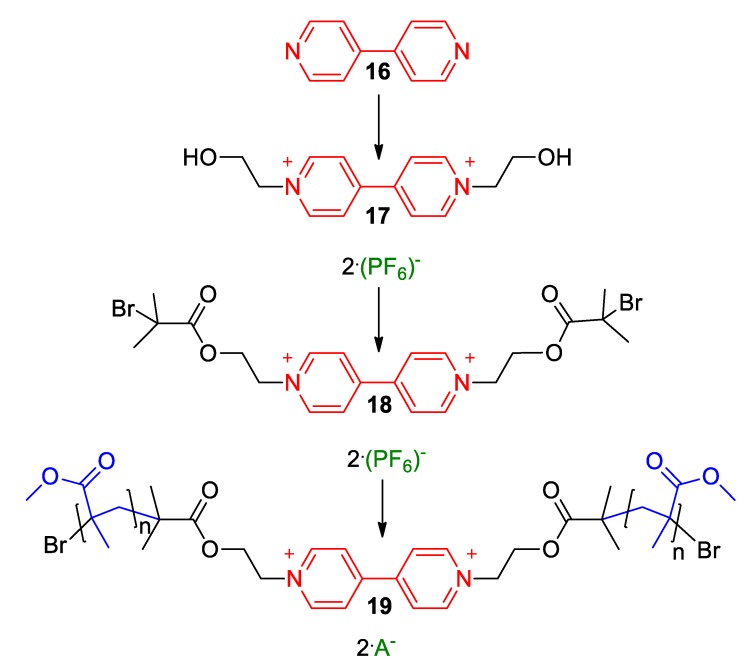
The three steps involving synthetic route to polymer **19** by Wang and Tsarevsky [43].

On the other hand, Wang and Tsarevsky recently managed to synthesize and characterize another 4,4′-bipyridine-involving polymer [43]. Specifically, their poly(methyl methacrylate)-based polymers involves only one viologen unit in the middle of the polymeric chain (Figure 8). In order to achieve that, they employed the synthetic route depicted in Figure 8. The key step of the synthesis (polymerization) was achieved via copper-mediated atom-transfer radical polymerization (ATRP). Importantly, the reaction conditions were chosen carefully in order to circumvent the undesired redox side-reactions, which create radicals (including viologen radical cations). The procedure was rendered successful under low concentrations of the Cu^I^ ATRP activator and avoiding external reducing agents.

In addition to the highly challenging synthetic work presented in their work, Wang and Tsarevsky also reported on the solvatochromism of the obtained polymers and emphasized on the shifts of the fluorescence maxima of the polymers in solvents of different polarity (the corresponding phenomenon is often called fluorosolvatochromism [34]). In particular, the viologen polymer **19** was found to fluoresce in solution; yet, it exhibits different emission spectra depending on the solvent. When photoexcited at 350 nm, only one band appeared in the emission spectra (emission spectra of the polymer were recorded in each of the solvents: chloroform, acetic acid, acetonitrile, or ethyl acetate). The band was centered at around 410 nm. However, when recorded in the solvents toluene, tetrahydrofurane (THF), vinyl acetate, DMF, or DMSO, the same polymer exhibited an additional fluorescence band significantly blue-shifted with respect to the 410 nm band (centered at around 540 nm). The color of the emitted light observed was thus blue-green. The authors attributed the emission of higher energy (410 nm/3.02 eV) to the planarized conformer of the viologen, i.e., the conformer having coplanar both pyridine rings [43]. In turn, the secondary band of lower energy (540 nm or 2.30 eV) having lower emission intensity was attributed to a twisted viologen conformation. This differentiation between solvents was further rationalized in terms of the aptitude of solvents to strongly interact with the dicationic viologen unit. Aprotic polar solvents such as DMSO, DMF, and THF are known to stabilize cations and arene solvents, e.g., toluene or vinyl acetate can interact with the π-conjugated viologen scaffold. It was concluded that this effect could be exploited for the sensing of arenes of high interest, e.g., explosive compounds such as nitroarenes [43].

**Figure 9:**
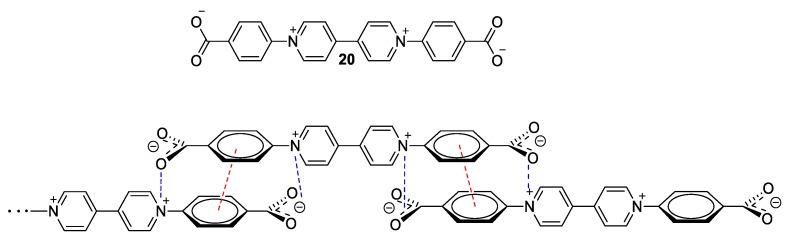
(**Top**) The zwitterionic viologen by Sui et al. and (**bottom**) the interactions observed in the crystal state (red dashed line, π–π stacking interactions; blue dashed lines, donor–acceptor interactions) [44].

Another example of a viologen with “direct” (solvato)chromic behavior was designed and synthesized by Sui et al. (Figure 9) [44]. Molecule **20** is π-conjugated and zwitterionic, comprising the bipyridinium dication and two para-benzoate units as functional groups of the two *N*-atoms. The compound does not involve any “external” anion. In the crystalline state, the zwitterionic viologen units build a very interesting supramolecular architecture, which is stabilized by extended π–π stacking of the phenyl rings of benzoate units between zwitterions lying parallel to one another as well as through anion–cation (electrostatic) interactions between carboxylates and quaternary nitrogen atoms of the viologens. Noteworthy, while the described crystal has a yellow color at ambient pressure, it becomes red under high pressure (2.8 GPa) and green right after decompression but regains its initial yellow color after remaining at ambient pressure for some time. The yellow color attributed to a charge transfer (CT) band (UV–vis diffuse reflectance spectra at around 400 nm extending to roughly 470 nm). This CT absorption hints that CT can occur from the carboxylate units to the cationic viologen units even at ambient pressure. However, compression brings the parallel-lying zwitterionic molecules closer and this has a drastic effect on the CT energy, which is thus lowered. The green color obtained after decompression is attributed to four absorption bands appearing in the visible diffuse reflectance spectra (480, 610, 670, and 740 nm). This reversible piezochromic behavior, which is clearly associated with dramatic electronic changes upon compression/decompression of the crystals of **20**, is not the only feature of this viologen-based compound. CT can also be highly affected by dehydration. Specifically, the crystals become green upon loss of water and the crystal structure changes significantly (dehydration was tested at temperatures up to 250 °C). The dehydration apparently affects the CT in a drastic way from the carboxylate to the viologen, and the electronic properties of dehydrated crystals (green) are very similar to the crystals obtained after compression (also green). The latter effect is not often observed and it resembles a very interesting case of supramolecular solvatochromism [45].

**Figure 10:**
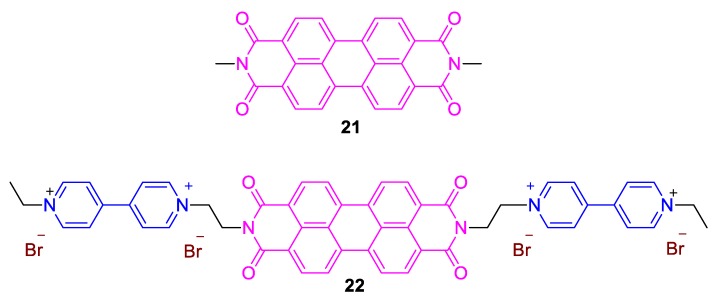
Structure of the perylene diimide-based fluorescent viologen **22** by Pramanik et al. [46] and perylene diimide precursor (**21**).

Pramanik et al., on the other hand, very recently reported on the preparation of a conjugate involving two viologen units (compound **22**) and a bridging perylenediimide unit (Figure 10, **21**) [46]. The viologen-based compound (Figure 10) was found to exhibit a marked solvatochromic and base-sensitive behavior. The combination of the strong electron-withdrawing and base-sensitive viologen moieties along with the emissive perylenediimide group significantly decreases the detection limit of various bases (e.g., NaOH, Et_3_N, piperidine, N,N-dimethyl-4-aminopyridine, pyridine, triisopropylamine, etc.) to the ppb level and thus renders this chromic viologen an extremely sensitive compound to bases. Noteworthy, the base-sensitive character of various viologens is strongly associated with their aptitude to form charge transfer complexes with a wide range of electron-rich compounds like phenols, TTF, etc. [21,22,23]. The base-sensitive character is also observed when solid forms (e.g., crystals) of the conjugate are exposed to vapors of various volatile bases, and this could be very useful for the development of base-sensitive technologies. Regarding the solvatochromic behavior of the conjugate, it was found that there is a fairly good linear correlation between the emission and absorption maxima wavenumbers and Reichardt’s solvent polarity scale (E_T_(30)) [34]. The correlation is better in case of absorbance compared to emission spectra; however, in both cases, a decrease of the transition energy when solvent polarity increases is observed. This corresponds to a positive solvatochromic behavior [41].

**Figure 11:**
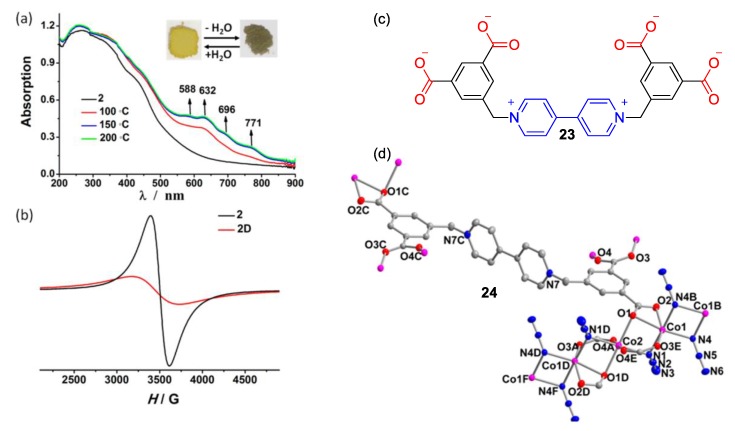
(**a**) UV−vis reflectance spectra (inset depicts corresponding photographs of the solid), and (**b**) EPR spectra of compound **24** prior to and after dehydration. (**c**) The structure of viologen backbone (**23**) utilized in **24**(**d**) partial crystal structure of the chromic complex **24**. Reproduced with permission from [47]. Copyright American Chemical Society.

Gong et al., on the other hand, utilized N,N′-bis(3,5-dicarboxylatobenzyl)-4,4′-bipyridinium (**23**) as a multifunctional linker in a metal−organic framework (MOF) involving Co or Mn (see structure **24**, Figure 11d) [47]. The viologen scaffold/linker not only assisted in the stabilization of the MOF, but it further offered a multifunctional character to the material that was found to exhibit photo- and hydro-chromism. Noteworthy, the viologen zwitterions utilized by Gong et al. were very similar to the ones utilized in the work by Yang et al. (vide supra Figure 9). The photochromism on one hand is attributed to the efficient electron transfer and the formation of radicals induced by light. As in numerous other examples, the electron-deficient character of viologens assist such phenomena [1,16,18]. Moreover, the MOFs were found to be sensitive to water undergoing a reversible color change when adsorbing water in the solid state, and these changes could be easily observed by naked-eye (see Figure 11a) but were also monitored through UV−Vis reflectance spectra. Importantly, drastic changes in the electron paramagnetic resonance spectra of the MOFs were observed during the hydration−dehydration cycles. These very distinct changes (see Figure 11b) were attributed to the magnetic interactions between the radical species formed upon dehydration and the metal ions of the MOFs.

**Figure 12:**
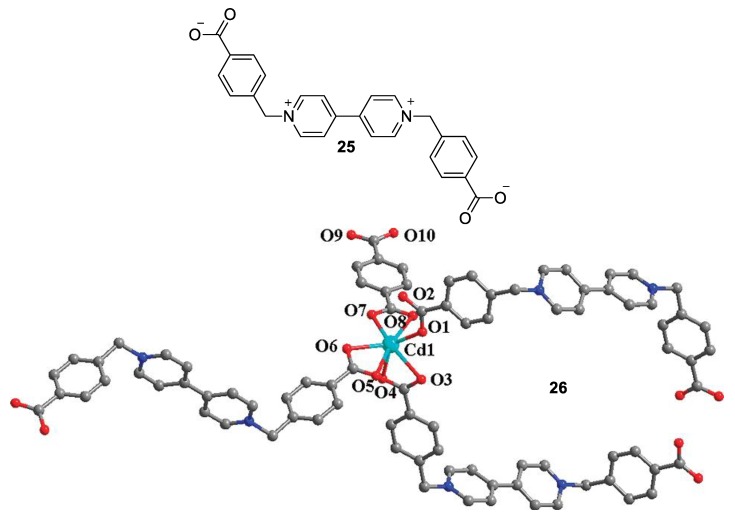
The zwitterionic viologen utilized by Yao et al. (**25**) and the structure of the repeated Cd (II) complex (**26**) involving viologen 25. Reproduced with permission from [49]. Copyright American Chemical Society.

In a similar fashion, two more research groups utilized a viologen bearing flexible benzyl-based N-substituents with only one carboxylate unit each to obtain a Zn7-cluster/MOF with interesting multi-chromic properties (electrochromic, thermochromic, photochromic, and basochromic) [48] and a Cd(II) 3D polypseudorotaxane array with photo- and thermo-chromic properties (see viologen **25** and structure **26**, Figure 12) [49].

Even though this last example involves metal ions, the chromic behavior of the final materials (MOFs) is mainly associated with the unique properties of viologens. In this last example, there is no coordination of metal ions with the 4,4′-bipyridine *N*-atoms of the MOFs. In the next paragraph, important recent paradigms of chromic compounds involving 4,4′-bipyridine and metal ions in coordination are reviewed.

## 4. Solvatochromic Complexes Involving Monoquats as Ligands

**Figure 13:**
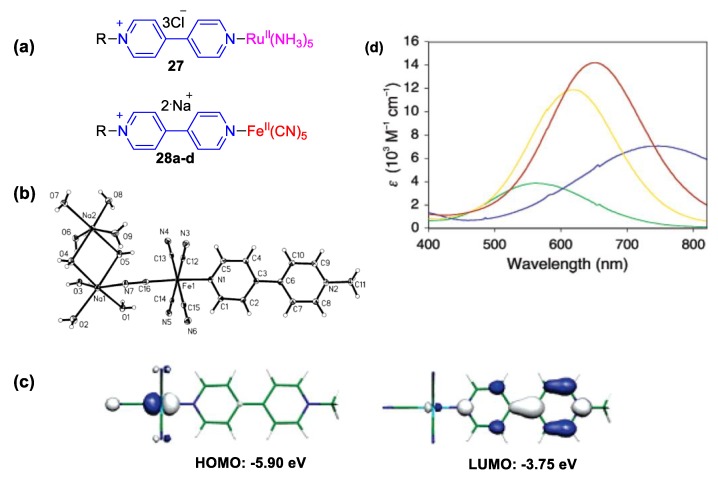
(**a**) Structure of the pentacyanoferrate(II) complexes (**28a**–**28d**) by Coe et al. [55] **(28a: R = Me; 28b**: R = Ph; **28c**: R = 4-AcPh; **28d**: R = 2-Pym) and (**b**) crystal structure of complex **28a**. (**c**) HOMO and LUMO representations and corresponding energies for **28a** calculated on the PCM(H_2_O)-B3P86/6-311 + G* level of theory. (**d**) UV–Vis spectra of **27** and **28b** recorded in water (yellow: **27**; green: **28b**) and MeOH (red: **27**; blue: **28b**)). Reproduced with permission from [55]. Copyright American Chemical Society.

Monoquaternized 4,4′-bipyridines, also called monoquats, are often used as ligands in a wide range of transition metal complexes. When the metal center coordinated by a monoquat ligand exhibits two redox states and lies in the lowest redox state, the occurrence of intramolecular charge transfer from the metal center to the monoquat is possible via absorption of light (a phenomenon known as metal-to-ligand charge transfer (MLCT)). For many corresponding complexes of Fe^II^, Ru^II^, Co^II^, and Os^II^, the energy of light is relatively low and MLCT can occur merely by absorption of visible light. Hence, these complexes have vivid colors both in the solid state and in solution phase. Pyridine-involving complexes of the aforementioned transition metals have been thoroughly studied for many years and it is known that they exhibit visible MLCT bands, which are, in many cases, drastically affected by solvent polarity changes. Blandamer et al. [50] as well as Toma and Takasugi [51] observed that solvent polarity has a drastic effect on the electronic spectra of prentacyanoferrate (II) complexes bearing pyridine-based ligands. Pinheiro et al. [52] studied an intensely solvatochromic pentacyano ruthenium(II) complex with a 2,2′-bipyridine ligand and exploited its particularly high sensitivity to traces of water for its detection in aprotic solvents using merely UV–Vis spectrophotometry [47]. Dyes of this family have been used as solvatochromic probes [53,54]. Some studies by Coe et al. brought 4,4′-bipyridine ligands to the fore [55]. Pentaaminoruthenium(II) (**27**) and pentacyanoferrate(II) (**28a**–**28d**) complexes involving monoquats as ligands (see Figure 13a) were found to exhibit intense solvatochromism featuring dramatic changes in their electronic spectra when moving from water to MeOH (Figure 13b). Even though the latter is a solvent with small difference in polarity when compared to water, shifts of the MLCT band of even 236 nm were observed (see Table 1). Compounds **28a**–**28d** also exhibit non-linear optical properties, and this renders these compounds promising candidates for modern high-tech applications [55]. Additionally, the same research group managed to crystallize complex **28a** and solve its structure (Figure 13b). This is probably the only published crystal structure of a pentacyanoferrate (II) complex involving a monoquat ligand (see Figure 13b). The highly hygroscopic character of these complexes renders the crystallization difficult. Indeed, the crystal structure encompasses a large number of water molecules. Interestingly, the dihedral angle between the two pryridine rings was determined to be 24.26°, and, given this finding, no significant charge transfer in the electronic ground state can be expected. Nonetheless, the solvatochromic responses of **28a** are drastic. The substituent on the quaternary N-atom of the ligand was proved to demonstrate an important role on the electronic properties of the complexes **28a**–**28d** (see also Table 1). The solvatochromism followed the intensity sequence: **28d** > **28c** > **28b** > **28a** [55,56]. Quantum chemical calculations indicated that pentacyanoferrate(II) plays the role of the electron donor and the monoquat that of the strong electron acceptor (see Lowest Unoccupied Molecular Orbital (LUMO) and Highest Occupied Molecular Orbital (HOMO) of compound **28a**, Figure 13c) [55]. The solvatochromic responses of the ruthenium complex **27** was of moderate intensity when compared with the anionic penatacyanoferrate(II) analog **28b**.

**Figure 14:**
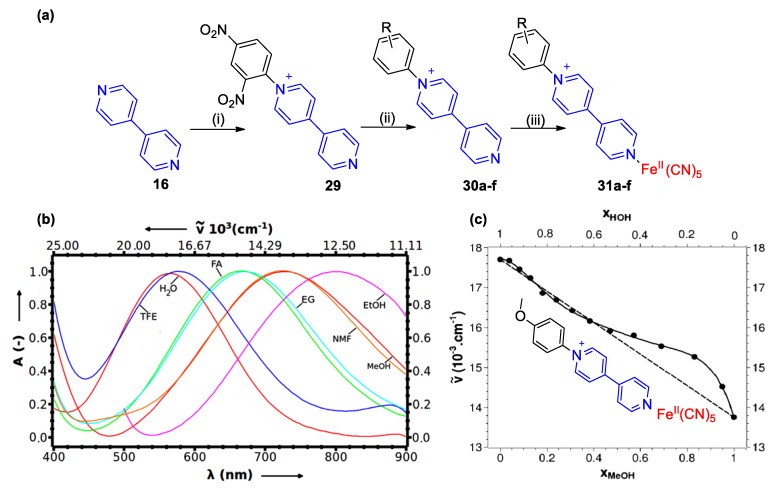
(**a**) Synthetic route followed by Papadakis and Tsolomitis for the synthesis of solvatochromicmonoquat-based complexes **31a**–**f** (**31a**: R = *p*-OMe; **31b**: R= *p*-Me; **31c**: R= *m*-Me; **31d**: R = *p*-Cl; **31e**: R = *p*-Br; **31f**: R = *p*-CN). (i) Dinitrochlorobenzene, EtOH (reflux); (ii) aniline R-Ph-NH_2_, EtOH(reflux); (iii) Na_3_[Fe^II^(CN)_5_NH_3_] water, r.t., then EtOH 4 °C. (**b**) Partial visible spectra (MLCT band) of **31a** recorded in different solvents: bathochromism with decrease of solvent polarity. (**c**) Plot of measured solvatochromic shifts (MLCT maxima wavenumbers of **31a**) in aqueous MeOH versus mol ratio of MeOH, Theideallineis represented asdashed line. Reproduced with permission from [54]. Copyright Springer.

In a similar fashion, Papadakis and Tsolomitis utilized Zincke reaction to produce a number of monoquats in which the N-substituent was an aryl group (see Figure 14a) [53]. They then utilized these monoquats as ligands and synthesized six pentacyanoferrate(II) complexes, which were found to exhibit intense solvatochromism. The role of the aryl substituent in these complexes is drastic. Strong electron accepting substituents (e.g., para-CN) shift substantially the MLCT bands of the complexes to even longer wavelengths. Practically, it was shown that in those cases and when in less polar solvents (MeOH or EtOH), MLCT bands could partly enter the near infrared region of the electromagnetic spectrum (see Figure 14b). The MLCT energy differences between water and MeOH of complexes **31a**–**f** were similar to the ones of complexes **28a**–**28d** by Coe et al. [55] (see Table 1).

Due to their intense solvatochromic behavior clearly associated with the presence of the monoquat ligands, complexes **31a**–**31f** can probe small solvent polarity changes like those induced when altering the solvent/cosolvent ratio in binary solvent mixture (BSM). Papadakis et al. showed that preferential solvation phenomena as well as specific local solvation effects can be conveniently investigated with the use of compounds **31a**–**31f** [54,57]. Indeed, even BSMs involving very similar solvents in terms of polarity, e.g., water and formamide, or water and ethylene glycol were studied [54,57]. A characteristic response to solvent polarity and preferential solvation effects occurring in water/MeOH mixtures are shown in Figure 14c. Due to their large solvatochromic shifts and their high solubilities in polar organic solvents and water, complexes **31a**–**f** could be outstanding solvent polarity indicators and probes of solute-microenvironment effects, especially useful in cases of media of high polarity [53,54,55,57].

**Figure 15:**
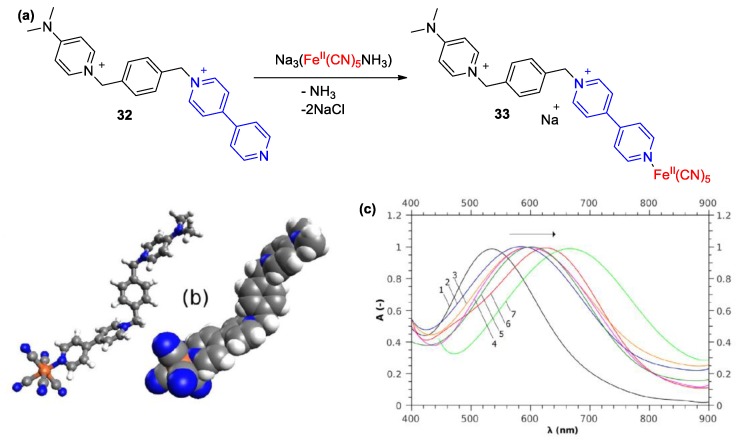
(**a**) Reaction scheme depicting the formation of solvatochromic complex **33** starting from the dicationic monoquat ligand **32**. (**b**) Calculated structure of a conformer of **33** (**grey: carbon atoms, white: hydrogen atoms; blue: nitrogen atoms; orange: iron atoms**. (**c**) Partial visible spectra (MLCT band) of **33** recorded in different solvents: bathochromism with decrease of solvent polarity. Reproduced with permission from [58]. Copyright Elsevier.

Moreover, a pentacyanoferrate(II) complex with a dicationic ligand bearing both a monoquat and a para-dimethylaminopyridinium unit (compounds **32**–**33**, Figure 15a,b) was synthesized and utilized in order to probe medium polarity effects in aqueous glucose [58]. A limitation of this complex was revealed since the complex was found to respond differently to neat solvents, BSMs, and aqueous glucose. In the latter case, the intensity of solvatochromism was substantially lower than that observed in neat solvents and this phenomenon was attributed to the lower degree of ionicity of the monoquat-involving Fe^II^ complex in aqueous glucose. Nevertheless, the intensity of solvatochromism in neat solvents remained in the same order of magnitude of other complexes of the same family (see Figure 15c). This interesting case of a 4,4′-bipyridine complex reflects the drastic role of this heterocyclic compound in the medium responsiveness of transition metal complexes.

**Figure 16:**
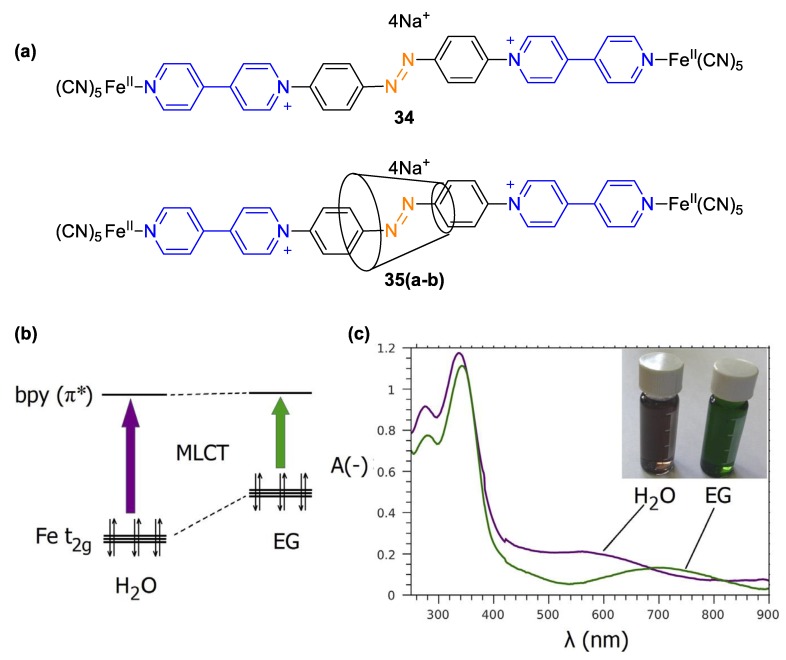
(**a**) Structure of [2]rotaxanes **35a**–**35b (35a**: α-cyclodextrin; **35b**: β-cyclodextrin) and their cyclodextrin-free precursor **34**. (**b**) Molecular orbital diagram depicting the energy gap between donor and acceptor (MLCT transition) in **34**, **35a**–**35b** when moving from water to ethylene glycol. (**c**) UV–Vis spectra of **35a** in neat water (purple line) and neat ethylene glycol (green line). Inset depicts the color of the two solutions. Reproduced with permission from [59]. Copyright Elsevier.

Pentacyanoferate(II) units have also been used for the efficient capping of [2]rotaxanes involving azo-bridged monoquat-based linear components and cyclodextrin (α- or β-) macrocycles (Figure 16, **34, 35a**–**35d**) [59]. Deligkiozi et al. exploited the combination of pentacyanoiron(II) and a linear 4,4′-bipyridine skeleton with extended π-conjugation. The resulting interlocked systems proved to be markedly solvatochromic as a result of the efficient push–pull effect along the extended π-conjugated linear skeleton. Their responses (MLCT solvatochromic shifts) to changes in the polarity of the medium (BSMs consisting of water and ethylene glycol and neat solvents thereof) were as high as 126 nm [60]. In addition to the solvatochromism associated to the CT from Fe (II) to the paraquat electron-deficient system, it was observed that the *n*–π* transitions of the azo-group were also responsive to solvent polarity changes but to a lesser extent. The bis-monoquat scaffold of these materials was used earlier in some analogous [2]rotaxanes by the same research group, but the stoppering groups were organic in that case: 2,4-dinitrophenyl groups [61]. These viologen-based [2]rotaxanes (**36**–**37**) were not nearly as solvatochromic compared to their pentacyanoferrate(II)-involving analogs but their vis-band associated to azo-transitions was found to be quite responsive to the inclusion environment provided by the cyclodextrins. This observation underlines the importance of combining the strong electron-deficient 4,4′-bipyridine backbone with a strong electron donor when designing complexes with desired high CT and solvatochromic performances. Specifically, shifts of 25 nm were observed when comparing the cyclodextrin-free precursor and any of the α- or β-cyclodextrin [2]rotaxanes. Similar environment-responsive effects have also been reported for [2]rotaxanes-involving viologen-like axial parts and cyclodextrins [62]. Moreover, these compounds readily undergo reversible *E*–*Z* photoisomerizations, which has been exploited in the development of polar molecular shuttles as well as photoconductive compounds [61,63]. Visible light irradiation of the compounds can drastically trigger conductivity responses. The role of the viologen backbone on this light-responsive behavior is essential [63,64]. Taken together, it could be thus stated that the 4,4′-bipyridine scaffold acts as a multifunctional platform that can yield different kinds of responses (to solvent polarity or light) depending on the capping (ending) groups.

**Figure 17:**
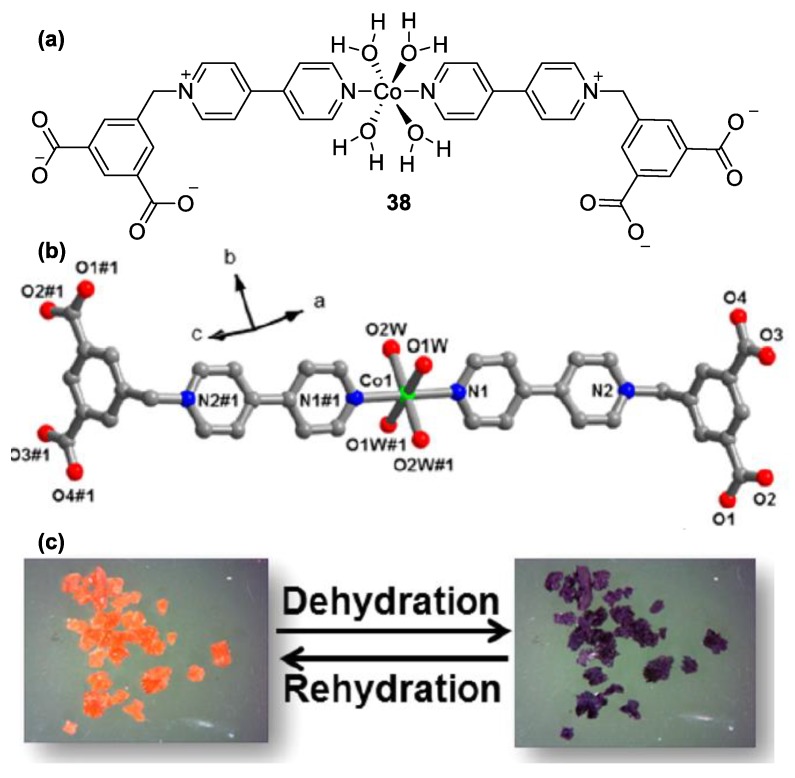
(**a**) Structure of complex **38**, (**b**) partial crystal structure of **38**, and (**c**) reversible color change of complex **38** upon dehydration/rehydration. Reproduced with permission from [65]. Copyright American Chemical Society.

There are various other examples of chromic complexes involving monoquat ligands. Very recently, Yang et al. reported on a multifunctional chromic Co(II) complex bearing a *N*-monosubstituted 4,4′-bipyridine ligand [65]. The aforementioned ligand contained two carboxylate entities (compound **38**, Figure 17a); however, ligation of the Co(II) ions was achieved by the pyridinic *N*-atom of the monoquat. The complex has an octahedral geometry with four coordinated water molecules and two monoquat units in the two apical positions. The carboxylate groups demonstrate an important role in the supramolecular packing of this crystalline complex. What renders this complex interesting is its outstandingly multifunctional performance. Various stimuli such as presence of a base (e.g., ammonia, ethenediamine, etc.) vacuum, solvent polarity, light, or heat can trigger sizable shifts in the absorption spectrum of **38**. This multifunctional performance is also associated with the high stability and reversibility of the chromic effects. All these characteristics render **38** a very important candidate for sensing applications of various analytes and environment conditions. Noteworthy is the reversible, dramatic color change of crystals of the complex from orange to violet upon dehydration (Figure 17c).

**Figure 18:**
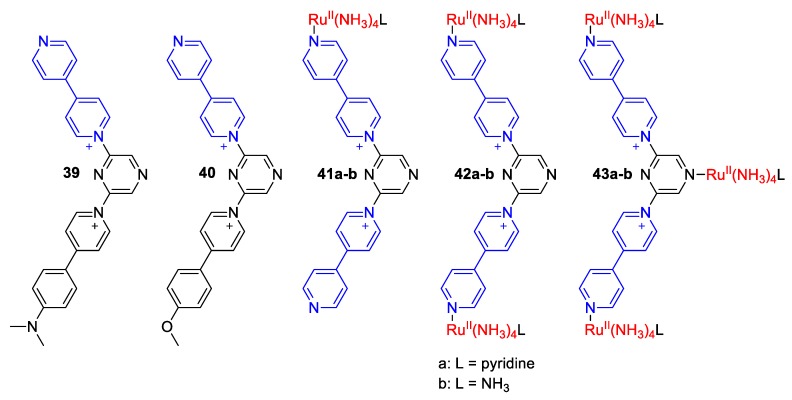
Structure of the monoquats **39**–**40** and Ru(II) complexes **41**–**43**(**a**–**b**) synthesized by Coe et al. [66,67].

Coe et al. prepared various bis-monoquat derivatives (**39**–**40**) [66] as well as Ru(II) complexes (**41**–**43**) [67] bearing a pyrazine bridge (Figure 18). All compounds were evaluated in terms of their non-linear optical (NLO) responses [66,67]. Due to their push–pull character (MeO–, Me_2_N–, or Ru(II) donor in π-conjugation with the strong accepting quaternary N-atoms of the monoquats), these complexes are potentially good candidates for chromic applications. This assumption is connected with both the NLO character of these compounds, a feature of which is solvatochromism [34] and the knowledge gained for similar complexes analyzed above.

## 5. Multicomponent Chromic Systems Involving Viologens

### 5.1. Glucose-Sensing Systems Involving Viologens

**Figure 19:**
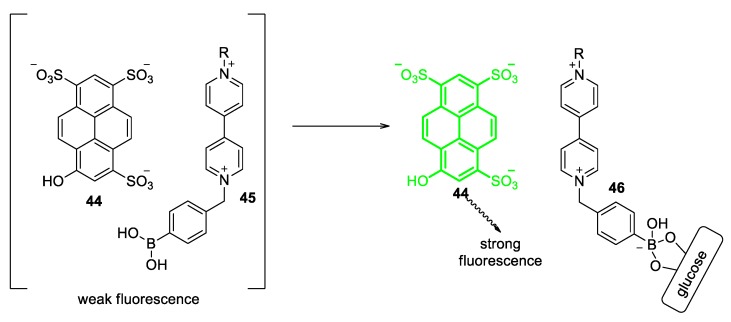
Scheme representing the quenching of pyranine (**44**) by a viologen (**45**) and the fluorescence recovery upon binding of glucose onto **45**.

The interaction of viologens with fluorescent compounds can lead to environment-sensitive systems and this has been largely exploited in the research of glucose-sensing [68]. Of primary interest are the ion pairs between viologens and pyranine (Compound **44**, Figure 19). Pyranine is a water-soluble pyrene-based fluorescent compound exhibiting intense fluorescence (λ_em_ = 650 nm) [69]. In the presence of a viologen, the fluorescence of pyranine is significantly weaker than that in the absence of a viologen and this comes as a result of the fact that viologens can act as fluorescence quenchers for pyranine. This effect is attributed to the ionic interaction between the anionic pyranine and a cationic viologen. In solution, the formation of an ion pair between those counterparts leads to a non-fluorescent electronic ground state [69].

**Figure 20:**
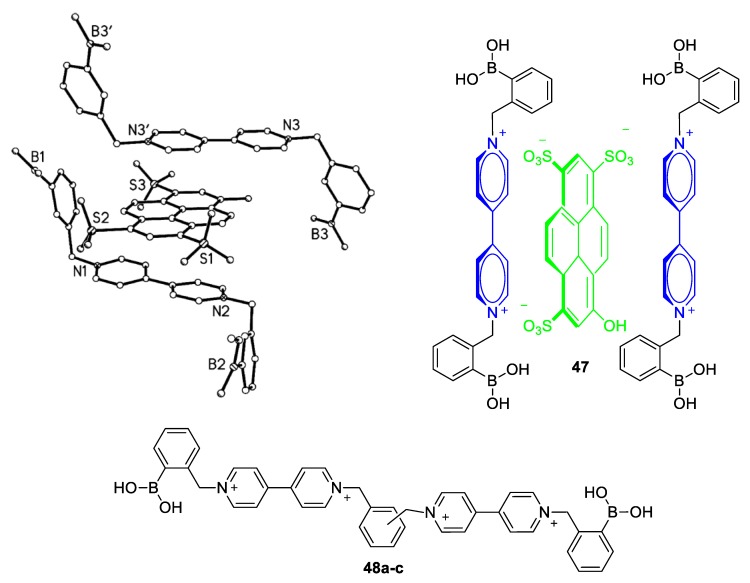
(**a**) Crystal structure of the ion pair **47** between pyranine and a symmetric viologen. (**b**) Structure representation of **47**. (**c**) Viologens **48a**–**48c** developed by Singaram and coworkers for efficient quenching of pyranine/glucose sensing (**48a**: ortho; **48b**: meta; **48c**: para). Reproduced with permission from [72]. Copyright American Chemical Society.

Upon incorporation of a boronic acid (BA) group (–B(OH)_2_) in a viologen, the latter retains its fluorescence quenching aptitude; yet, a new possibility for the pyranine–viologen ion pair is generated. Due to the high affinity of boronic acids to diols, polyalcohols, and monosaccharides (e.g., glucose), a boronic-acid-functionalized viologen can efficiently bind to such a substrate [70]. When this happens, disruption of the pyranine–viologen ion pair occurs and non-paired pyranine anions fluoresce strongly (see Scheme of Figure 19). The effect is drastically dependent on the concentration of the monosaccharide, hence, these viologen/pyranine ion pairs could serve as glucose-sensitive systems [71]. Indeed, this methodology has been exploited for the development of efficient glucose sensing systems. Up to date, a variety of boronic-acid-functionalized viologens have been synthesized in order to obtain a very efficient glucose sensor based on viologens. One of the first motivating studies in this research field is that by Singaram and coworkers (Figure 20a). The research group managed to grow crystals and analyze the structure of the ion-pair **47** consisting of pyranine and a symmetric viologen having two benzyl *N*-substituents that involve BA units (Figure 20b). Very interestingly, a donor−acceptor π-stacking arrangement of the molecules (pyranine and viologen) was observed [72]. The formation of this type of ion-pair in solution results in very weak fluorescence. Indeed, the system was proved to be an efficient sensor for glucose. Moreover, using this specific viologen selectivity toward glucose over other sugars (fructose and galactose) was accomplished [72]. The same research group further synthesized a large variety of viologens with different ratios of BA units/viologen units as well as with various positive charges. Some of such important viologens are depicted in Figure 20 (products **48a**–**48c**) [73,74].

**Figure 21:**
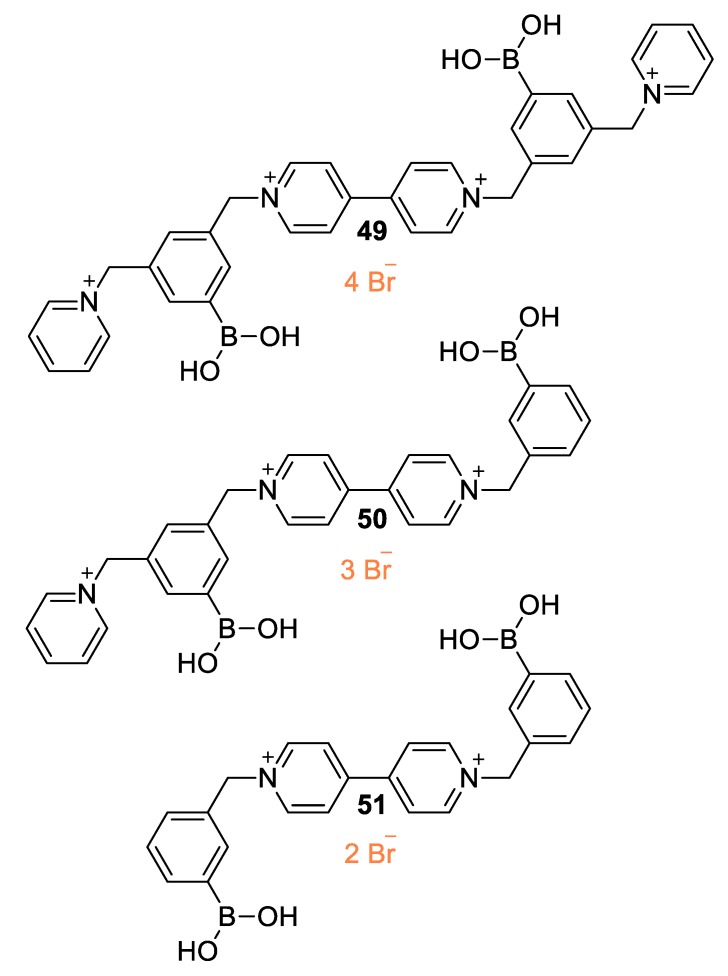
Three viologens with varying charge (**49**:4^+^; **50**:3^+^; **51**:2^+^) developed by Cordes et al. [76].

The effect of the charge of boronic acid-involving viologens toward pyranine fluorescence quenching was first reported by Cordes et al. (2005) [75]. Various substituted viologens were synthesized and their fluorescence quenching efficiencies were evaluated (Figure 21, viologens **49**–**51**). In all cases, the fluorescence of pyranine could be recovered in the presence of various monosaccharides with a strong dependency on monosaccharide concentration. Importantly, the charge of the various studied functionalized viologens drastically influenced both the quenching efficiency and the monosaccharide sensing aptitude and that was explained on the basis of the electrostatic interactions between the quencher and pyranine. Similar systems involving viologens developed by Gamsey et al. could have an aptitude for continuous glucose detection [76]. These immobilized systems involve viologens substituted with BA units within fluorescent poly (2-hydroxyethyl methacrylate) hydrogels [76]. This interesting approach is considered for usable sensing systems corresponding to glucose and other polyhydroxylated compounds [77].

### 5.2. Charge Transfer Complexes of Viologens

Charge transfer complexes (CTCs) constitute a class of complexes between electron-donating and electron-withdrawing species (molecules, ions, etc.) [78,79]. The dominating stabilizing force in these complexes is the electrostatic attraction between the donor and acceptor counterparts. Viologens have demonstrated a great role in this research field as their intense ionicity, electron-deficient character, and electro- and photo- activity as well as their versatility have been motivating for the development of novel CTCs with interesting properties [1,78]. Typically, CTCs are characterized by a distinct (often visible) band in their electronic spectra (CT band). Due to the dependency of the energy of CT on various parameters, e.g., light, solvent polarity, temperature, pressure, etc. and their often intense chromic behavior, CTCs are also considered nowadays as important candidates for sensing applications.

The investigation of ion pairing between the viologen dications and various counter anions is crucial for the development of environment-responsive systems (vide supra: viologen/pyranine pairs). Ion pairs involving viologen dications are prone to respond to even small changes in solvent polarity or other changes in their environment. Sailelli studied the ion pairing utilizing NMR and UV-Vis spectroscopies on 1,1′-di-*n*-octyl-4,4′-bipyridinium involving iodide counter anions in a group of organic solvents [80]. These viologen iodides exhibit distinct CT bands, the energy of which is readily affected by solvent polarity. Significant blue-shift of the CT bands was observed when solvent polarity increased. This phenomenon corresponds to negative solvatochromism [41]. More recently, Santos et al. reported on the photoinduced charge shifts in some viologen–tetraphenylborate complexes, which were studied by means of steady-state and time-resolved spectroscopic techniques [24]. An interesting reversible on–off photoswitching aptitude was observed in the solid state.

**Figure 22:**
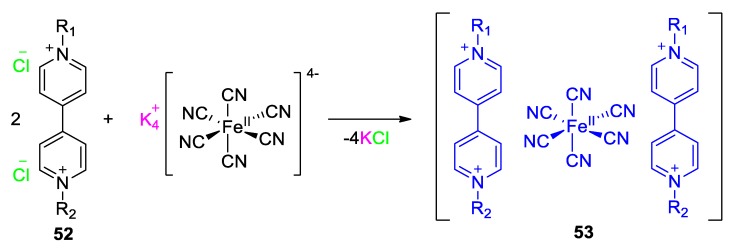
Scheme representing the formation of a charge transfer complex (CTC) between a viologen and ferrocyanide(II) anion. (CTC colored blue).

Perhaps the most popular CTCs between viologen cations and electron-donating anions are those involving hexacyanoferrate(II) anions [78]. The earliest example of such a viologen-involving CTC is the one reported by Nakahara and Wang in 1963 [81]. This CTC comprised of methylviologen cations (paraquat) and hexacyanoferrate anions (Figure 22, R_1_ = R_2_ = Me). The isolated complex is red-colored in the solid state, yet its aqueous solutions are red-purple with an absorption CT band situated at 540 nm [81,82]. It is noteworthy that different research groups realized early that the aforementioned band does not correspond to a viologen radical cation band as it differs in molar absorptivity, energy (radical cation band of methylviologen is centered at λ_max_ = 608 nm), as well as in shape when compared to the latter [83,84,85]. Members of the aforementioned class of CTCs exhibit interesting chromic phenomena and this was observed about 30 years ago by Hammack et al. [86]. The CTC of methylparaquat and hexacyanoferrate(II) anions can give piezochromic shifts as a response to the applied pressure [86]. The observed shifts were as high as 1700 cm^−1^ when the applied pressure was 10 kbar. This drastic change is clearly attributed to the effect of pressure on the relative distances between the electron donor (ferrocyanide(II)) and acceptor (viologen dication) counterparts of the CTC. In turn, such a change results in the alteration of the electrostatic interactions between those two counterparts and finally of the CT energy. (A very similar situation was described for the zwitterionic crystalline viologen by Sui et al. [44] vide supra). Later on, Monk et al. studied in depth the energetics and kinetics of the formation of such complexes [87] and reported useful information on this important type of CTC and very similar spectral results with Nakahara and Wang.

**Figure 23:**
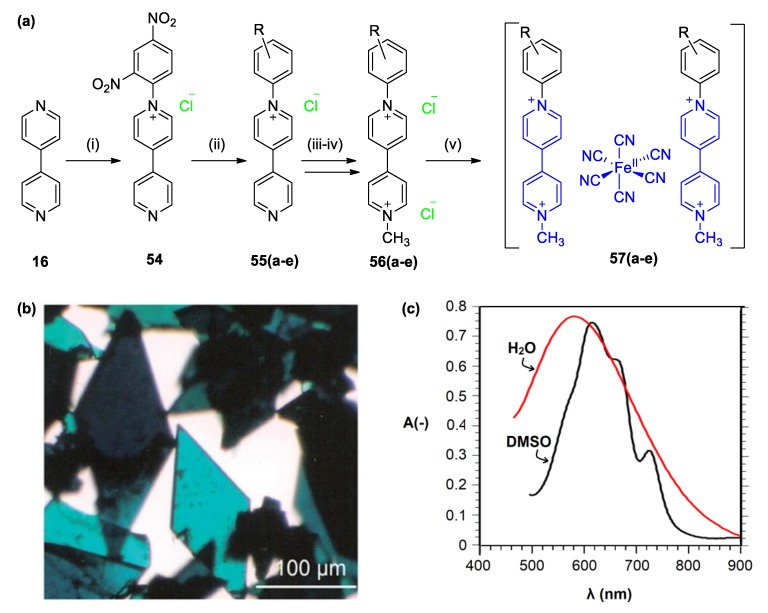
(**a**) Synthetic route followed by Papadakis et al. for the preparation of CTCs **57a**–**57e**. (i) Dinitrochlorobenzene, EtOH (reflux); (ii) aniline R-Ph-NH_2_, EtOH(reflux); (iii) MeI, MeOH reflux; (iv) NH_4_PF_6_, water then Et_4_NCl, acetone; (v) K_4_[Fe^II^(CN)_6_], water, r.t. (**b**) Photograph of **57a** crystals. (**c**) Partial visible spectra of **57a** recorded in water (red line) and DMSO (black line) (unpublished result by Papadakis et al.). Figure of panel (**b**) reproduced with permission from [88]. Copyright Royal Society of Chemistry.

More recently, various other research groups studied the effect of the viologen N-substituents on the CTC chromic performance (see various results on crystalline CTC of this family in Table 2). Abouelwafa et al. utilized aromatic N-substituents instead of aliphatic and reported that the corresponding supramolecular CTC with ferrocyanide(II) anion is photochromic and thermochromic [25]. CT was found to be enhanced upon light irradiation as indicated through various spectroscopic techniques including EPR and UV–vis spectroscopies [25]. More recently, Papadakis et al. synthesized a group of non-symmetric *N*-aryl, *N*′-methyl viologens and investigated the formation of CTCs with ferrocyanides (Figure 23a) [88]. In most of the cases, the colors of the formed CTCs were blue to green (Figure 23b). The role of the substituent on the CT energy and thus of the color of the CTCs was significant and followed a linear correlation with the Hammet’s substituent constant (*σ*) [88]. This indicated that there is “electronic communication” between the aryl ring and the bipyridinium skeleton, and, moreover, that strong electron accepting substituents stabilize the CTCs. What makes these supramolecular complexes even more interesting is that they are readily responsive to solvent polarity changes (see Figure 23c). A shift of approximately 40 nm was observed when moving from water to DMSO resulting in an apparent color change from blue to green. The electronic spectra of this CTC in these two solvents were also very different. While in water, the visible CTC band appears to be broad, exhibiting a single peak; whereas, in DMSO, the corresponding signal becomes less broad and four peaks are easily recognizable (at λ = 570, 619, 661, and 723 nm). The shape of this band is similar to that of a viologen radical cation. The solvatochromism of such a CTC has not been given much attention but constitutes an interesting chromic effect of the response of the complex to its environment.

**Figure 24:**
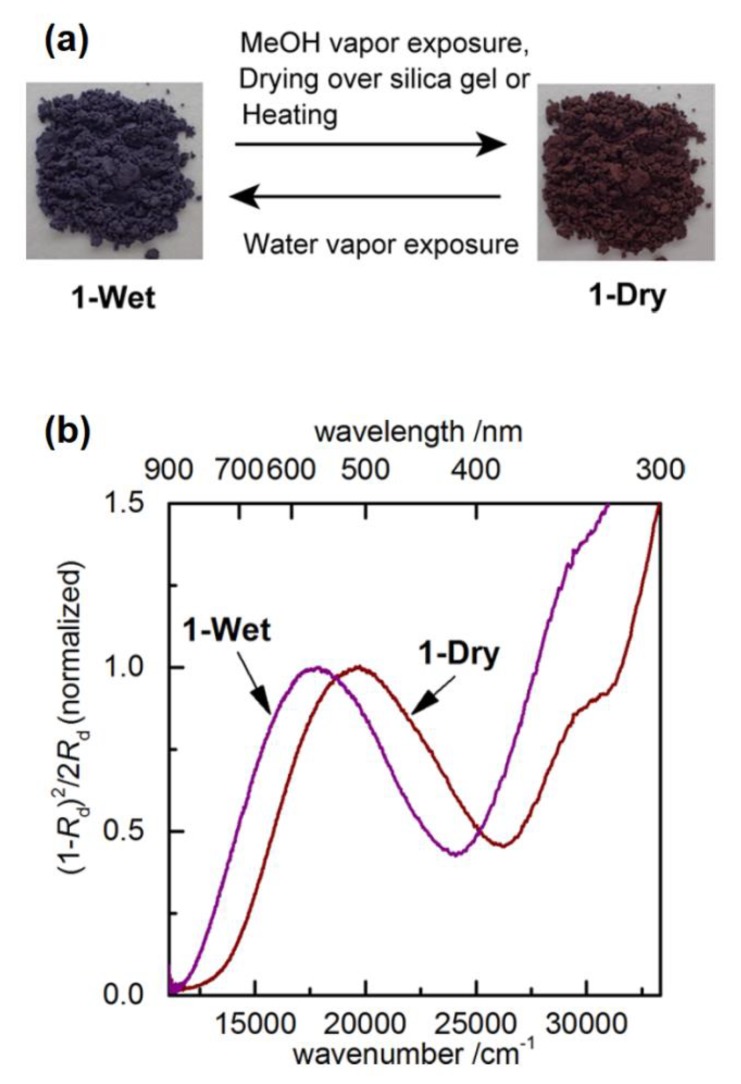
(**a**) Photographs depicting the reversible color change in a powder sample of CTC **53b** upon wetting and drying. (**b**) The bathochromic shift in the electronic spectra induced by wetting crystals of **53b**. Figures reproduced with permission from [91]. Copyright American Chemical Society.

Acknowledging the chromic behavior of this class of CTCs, Tanaka et al. recently reported on the reversible vapochromic and environment-sensitive character of crystalline CTCs between ethyl-viologen and hexacyanoferrate(II) anions [91]. The color of a dry sample of CTC **53b** (see Table 2) was found to be significantly different from that of a wet sample (see Figure 24a). This medium-responsive/vapochromic character is demonstrated through a drastic bathochromic shift in the UV/Vis solid-state diffuse reflective spectra when wetting (hydrating) a dry sample (Δλ = 53 nm: 560 nm for wet and 507 nm for dry sample; see Figure 24b). These visible bands are attributable to CT, and the one corresponding to the wet sample is very close to the value obtained for CTC **57a** by Papadakis et al., which is a CTC also involving a large amount of water molecules in crystal state (see Table 2).

The interpretation by Tanaka et al. for this intense chromic behavior is that removal of water molecules from a crystalline sample of **53b**, leaves back “bear” protons (H^+^), as counter cations which can be rehydrated to oxonium (OH_3_^+^) cations upon wetting. The protons are prone to hydrogen bonding with *N*-atoms of neighboring hexacyanoferrate (II) anions (–C≡N···H···N≡C–), which can induce changes in crystal packing, and which in turn influence the CT between hexacyanoferrate (II) anions and viologen dications.

**Figure 25:**
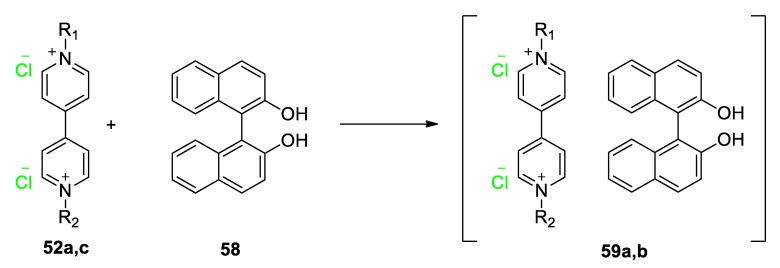
Reaction scheme describing the formation of CTCs of binaphthol **58** and viologens **52a** (R_1_ = R_2_ = Me) and **52c** (R_1_ = R_2_ = benzyl).

Various chromic CTCs of viologens involving other types of electron donors have also been reported. Kinuta et al. synthesized CTCs between two symmetric substituted viologens bearing chloride anions (**52a**,**52c**) and 1,10-bi-2-naphthol (**58**) [92]. The corresponding CTCs (**59a**,**59b**, Figure 25) are intensely thermochromic in the solid state being deep red-purple at room temperature and light red at temperatures as low as 80 K. The phenomenon exhibits reversibility, and it is attributed to the thermally sensitive nature of the crystal structure in which the distance between the viologen dications was found to intensely depend on the temperature. As mentioned above (zwitterionic crystalline viologen by Sui et al. [44] vide supra), crystal changes can influence the energy of CT and thus the color of the complexes and this leads to chromic CTCs. The study by Kinuta et al. is a very important example of materials with potential applications in solid-state supramolecular thermosensors.

### 5.3. Ionic Liquid Crystalline Systems Involving Viologens

Ionic liquid crystals (ILCs) constitute a blend of ionic liquids and liquid crystalline materials as denoted by their name [93]. Even though both classes of compounds/materials are known since the end of the 19th century [94,95], liquid crystals started gaining significant attention in the 1970s and ionic liquids in the 1990s when various products of both classes found some industrial applications [93]. ILCs have been recently given much attention, and pyridinium-based salts, particularly, viologens, have demonstrated an important role in the corresponding research field [96]. In this subfamily of ILCs, viologens serve as cationic backbones and are combined with suitable (weakly coordinating) anions such as PF_6_^−^, BPh_4_^−^ and many others [96]. Advances in this research field have been recently presented in a book pertaining to ILCs [97]. Herein, important examples of environment-responsive ILCs encompassing viologens and monoquats are reviewed.

**Figure 26:**
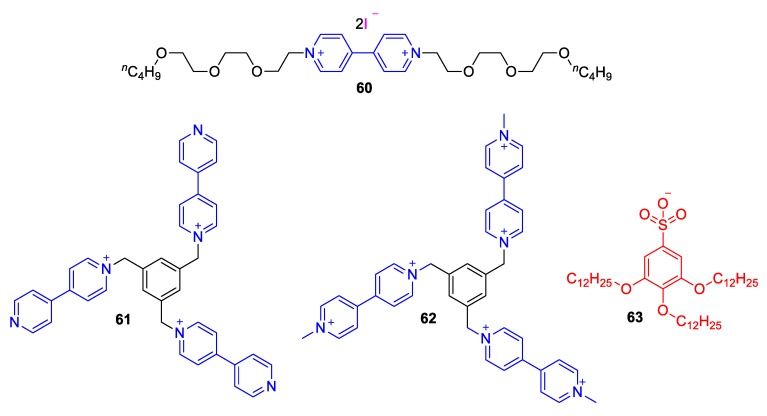
The viologen-involving ILCs developed by Tabushi et al. (**60**) **[98]** and Asaftei et al. (**61^3+^** and **62^6+^** bearing anion **63**) [99].

The first stimuli-sensitive ILC complex involving a viologen was reported by Tabushi et al. as early as 1986 [98]. The aforementioned complex composed of viologen **60** (see Figure 26), which was di-substituted with triethylene-oxide units bearing terminal *n*-butyl groups and involved iodine anions. This prototype semiconductive ILC system exhibits very interesting electrochromic properties and has been inspiring for a lot of other viologen-involving ILCs.

**Figure 27:**
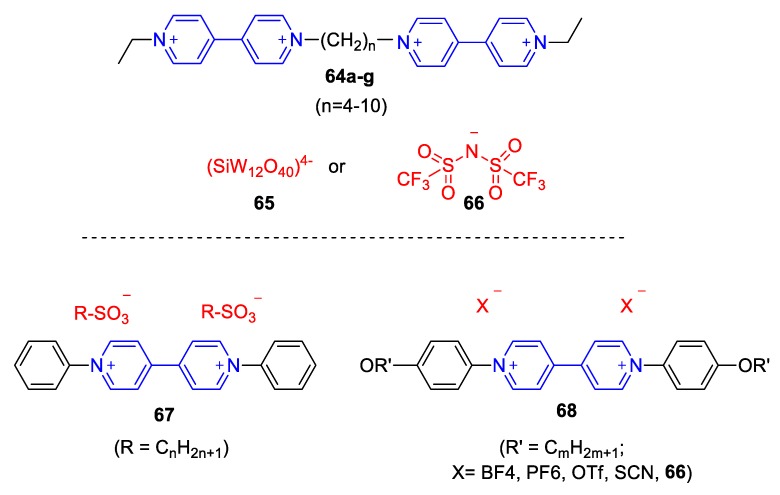
(**Top**) Viologen dimer cations and anions involved in the ILCs by Saielli and coworkers [100]. (**Bottom**) Diphenyl-viologens by Wang et al. [102].

More recently, Asaftei et al. synthesized some ILCs consisting of tripodal monoquat trications (**61**) and viologen hexacations (**62**). As anion, **63^−^** was employed [99]. The resulting ILCs exhibit a temperature-dependent electrochromic behavior. When methylviologen dication (**1^2+^**) was combined with the anion **63^−^,** a remarkable thermochromic performance was observed with a dramatic change from yellow to green-blue (absorption maximum λ_max_ = 600 nm) upon heating the sample above 75 °C. The heat-induced green-blue color maintained at 98% of its initial intensity at 25 °C for a day [99]. On the other hand, Saielli and coworkers reported on the thermal and electrical properties of ILs of a series of dimeric viologen salts (**64**, Figure 27) with (trifluoromethanesulfonyl)amide or dodecatungstosilicate anion, (SiW_12_O_40_)^4−^ [100]. It was concluded that the number of bridging methylene groups between the viologen units can readily influence the thermal properties of the final materials with even number of methylenes assisting the formation of stable crystalline phase even at high temperatures, while odd number of methylene groups leading to lower melting points compared to the previous and next members (bearing even number of –CH_2_–). This influential work has thus revealed a very interesting phenomenon, which illustrates the importance of structural design in the development of ILCs.

One of the rare cases of solvatochromic, viologen-based ILCs was developed by Gunaratne et al. [101]. The viologens employed, encompassing oligoether *N*,*N*′ substituents (in a similar fashion as in the prototype ILC **60** by Tabushi [98]) exhibit medium-responsiveness, as well as charge-transfer-complex formation aptitude. Wang et al. also reported a family of diphenyl-viologens (**67**,**68**, Figure 27) and investigated their ILCs employing a variety of anions [102]. Parallel to the interesting electrochemical properties, these ILCs found to exhibit solvatochromism. Specifically, the π–π* transition UV-bands of compounds **67** and **68** were situated at λ_max_ = 396 nm and 407 nm, respectively, when dissolved in CH_2_Cl_2_ but they were shifted to lower wavelengths (314 nm for **67**, 379–385 nm for **68**). These shifts correspond to negative solvatochromic responses (i.e., bathochromism as solvent polarity decreases) [41]. Interestingly, even though the MeOH or CH_2_Cl_2_ solutions of **68** exhibited no photoluminescence, they were found to emit light when dissolved in aqueous mixtures, especially at high water fractions (λ_em_ = 463 to 529 nm strongly depending on the counter anion). This effect was attributed to the aggregation occurring primarily in water-rich mixtures [102].

More recently, a multi-functional ILC material with aptitude to form bicontinuous cubic phases, based on a zwitterionic viologen was developed by Kobayashi and Ichikawa [103]. The amphiphilic zwitterionic viologen encompasses an ionic moiety and readily forms a 3D structure consisting of viologen layers. In their ionic state, the viologen units of the material are capable of undergoing one-electron reductions leading to a color change from colorless to purple (indicative of the formation of a radical cation (see also Figure 2), and this process can be triggered photochemically without disrupting the 3D structure [103].

Overall, this innovative research field is gaining more and more attraction and multi-responsive viologen ILC systems are becoming more abundant in the literature, a fact that is clearly associated with the multifunctional role of viologens [1,10,11,104].

## 6. Conclusions

Recent advances in the field of medium- and environment-responsive mono- and di-quaternized 4,4′-bipyridine derivatives have been reviewed. Emphasis has been placed on “direct” solvatochromic and generally chromic derivatives including compounds of small molecular weight as well as polymeric viologens and monoquat-involving materials. An important case of sensing application, i.e., glucose-sensing with the use of viologens was also reviewed. Moreover, the role of viologens in CTCs as well as in liquid crystalline systems was reviewed with an emphasis on compounds/materials exhibiting intense dependency on solvent polarity, temperature, or other environment inputs. Overall, the ionic and highly electron-deficient character of viologens and monoquats are the common characteristics for all reviewed cases of chromic and environment-sensitive derivatives. The properties (e.g., electronic, thermal, optical, photochemical, etc.) of the final materials/compounds can be efficiently tailored by employing the right *N*-substituents as well as the suitable counter anions. This “tunability” of the mono- and di-quaternized derivatives of 4,4′-bipyridine renders this family of compounds indispensable for novel functional materials with high responsiveness/sensitivity and external-control opportunities.

## Figures and Tables

**Table 1:** Solvatochromic shifts of the metal-to-ligand charge transfer (MLCT) bands of published monoquat ligated pentacyanoferrates.

Compound	R	λ_max_ (E_MLCT_) ^1^ in H_2_O	λ_max_ (E_MLCT_) ^1^ in MeOH	Δλ_max_ (ΔΕ_MLCT_) ^1^	Ref.
**28a**	Me	534 (2.32)	686 (1.81)	152 (0.51)	[55]
**28b**	Ph	566 (2.19)	738 (1.68)	172 (0.51)	[55]
**28c**	4-AcPh	584 (2.12)	771 (1.61)	187 (0.51)	[55]
**28d**	2-Pym	618 (2.00)	854 (1.45)	236 (0.55)	[55]
**31a**	4-OMePh	565 (2.19)	727 (1.71)	162 (0.48)	[53]
**31b**	4-MePh	563 (2.20)	730 (1.70)	167 (0.50)	[53]
**31c**	3-MePh	563 (2.20)	731 (1.70)	168 (0.50)	[53]
**31d**	4-ClPh	572 (2.17)	741 (1.67)	169 (0.50)	[53]
**31e**	4-BrPh	573 (2.16)	741 (1.67)	168 (0.49)	[53]
**31f**	4-CNPh	586 (2.12)	755 (1.64)	169 (0.48)	[53]

^1^ λ_max_ values in nm and E_MLCT_ values in eV.

**Table 2:** Geometric characteristics (crystallographic) and spectral data for various known CTCs of viologens with ferrocyanide (II) anion.

CTC	R_1_, R_2_^1^	*φ* (^ο^) ^2^	*R*_CC_ (Å) ^3^	*R*_pyFe_*^m^* (Å) ^4^	λ_max_ (nm) ^5^	Ref.
**53a**	Me, Me	37.4	1.481	5.603	540	[89]
**53b**	Et, Et	1.30	1.481	6.321	–	[90]
**53b**	Et, Et	15.6	1.476	6.137	507,560	[91]
**57a**	*p*-tolyl, Me (1)	10.3	1.434	5.767	574	[88]
**57a**	*p*-tolyl, Me (2)	27.6	1.480	5.604	574	[88]
**53c**	2,4-(NO_2_)_2_Ph, 2,4-(NO_2_)_2_Ph	13.8	1.480	5.520	420, 532	[25]

^1^ The two N substituents of viologen dications. ^2^ Dihedral angle between the two pyridine rings of the viologens. ^3^ Distance between C-atoms connecting the two pyridine rings. ^4^ Minimal distance between the centroid of closest pyridinium ring to a ferrocyanide anion. ^5^ Absorption maximum wavelength of the visible CTC band measured in water.

## References

[1] Monk P.M.S. (1998). The Viologens: Physicochemical Properties, Synthesis and Applications of the Salts of 4,4-Bipyridine.

[2] Baroncini M., Silvi S., Credi A. (2019). Photo- and Redox-Driven Artificial Molecular Motors. Chem. Rev..

[3] Wang Y., Frasconi M., Stoddart J.F. (2017). Introducing Stable Radicals into Molecular Machines. ACS Cent. Sci..

[4] Erbas-Cakmak S., Leigh D.A., McTernan C.T., Nussbaumer A.L. (2015). Artificial Molecular Machines. Chem. Rev..

[5] Fahrenbach A.C., Zhu Z., Cao D., Liu W.-G., Li H., Dey S.K., Basu S., Trabolsi A., Botros Y.Y., Goddard III W.A. (2012). Radically Enhanced Molecular Switches. J. Am. Chem. Soc..

[6] Feringa B.L., Browne W.R. (2011). Molecular Switches.

[7] Škorjanc T., Shetty D., Olson M.A., Trabolsi A. (2019). Design Strategies and Redox-Dependent Applications of Insoluble Viologen-Based Covalent Organic Polymers. ACS App. Mater. Inter..

[8] Aulakh D., Varghese J.R., Wriedt M. (2015). A New Design Strategy to Access Zwitterionic Metal–Organic Frameworks from Anionic Viologen Derivates. Inorg. Chem..

[9] Li P., Zhou L.-J., Yang N.-N., Sui Q., Gong T., Gao E.-Q. (2018). Metal–Organic Frameworks with Extended Viologen Units: Metal-Dependent Photochromism, Photomodulable Fluorescence, and Sensing Properties. Cryst. Growth Des..

[10] Striepe L., Baumgartner T. (2017). Viologens and Their Application as Functional Materials. Chem. Eur. J..

[11] Ding J., Zheng C., Wang L., Lu C., Zhang B., Chen Y., Li M., Zhai G., Zhuang X. (2019). Viologen-inspired functional materials: Synthetic strategies and applications. J. Mater. Chem. A.

[12] Shah K.W., Wang S.-X., Xiang D., Soo Y., Xu J. (2019). Viologen-Based Electrochromic Materials: From Small Molecules, Polymers and Composites to Their Applications. Polymers.

[13] Bird C.L., Kuhn A.T. (1981). Electrochemistry of the viologens. Chem. Soc. Rev..

[14] Škorjanc T., Shetty D., Olson M.A., Trabolsi A. (2019). Viologen-based electrochromic materials and devices. J. Mater. Chem. C.

[15] Murugavel K. (2014). Benzylic viologen dendrimers: A review of their synthesis, properties and applications. Polym. Chem..

[16] Monk P., Mortimer R., Rosseinsky D. (2007). Electrochromism and Electrochromic Devices.

[17] Benniston A.C., Harriman A., Li P., Rostron J.P., Harrington R.W., Clegg W. (2007). A Spectroscopic Study of the Reduction of Geometrically Restrained Viologens. Chem. Eur. J..

[18] Crano J.C., Guglielmetti R.J. (2002). Organic photochromic and thermochromic compounds. Main Photochromic Families.

[19] Tsukahara K., Wilkins R.G. (1985). Kinetics of reduction of eight viologens by dithionite ion. J. Am. Chem. Soc..

[20] Fiala T., Ludvíková L., Heger D., Švec J., Slanina T., Vetráková L., Babiak M., Nečas M., Kulhánek P., Klán P. (2017). Bambusuril as a One-Electron Donor for Photoinduced Electron Transfer to Methyl Viologen in Mixed Crystals. J. Am. Chem. Soc..

[21] Simonsen K.B., Becher J., Thorup N., Cava M.P. (1998). Synthesis and X-ray crystal structure of the first tetrathiafulvalene-based acceptor–donor–acceptor sandwich. Chem. Commun..

[22] Ponnu A., Sung J., Spears K.G. (2006). Ultrafast Electron-Transfer and Solvent Adiabaticity Effects in Viologen Charge-Transfer Complexes. J. Phys. Chem. A.

[23] Hwang H.J., Lee S.K., Lee S., Park J.W. (1999). An NMR study on the conformation of naphthalene–viologen linked compounds: Effect of flexible spacer length. J. Chem. Soc. Perkin Trans..

[24] Santos W.G., Budkina D.S., Deflon V.M., Tarnovsky A.N., Cardoso D.R., Forbes M.D.E. (2017). Photoinduced Charge Shifts and Electron Transfer in Viologen–Tetraphenylborate Complexes: Push–Pull Character of the Exciplex. J. Am. Chem. Soc..

[25] Abouelwafa A.S., Mereacre V., Balaban T.S., Anson C.E., Powell A.K. (2010). Photo- and thermally-enhanced charge separation in supramolecular viologen–hexacyanoferrate complexes. Cryst. Eng. Comm..

[26] Mau A.W.-H., Overbeek J.M., Loder J.W., Sasse W.H.F. (1986). On the fluorescence and photoreduction of methyl viologens. J. Chem. Soc. Faraday Trans..

[27] Alvaro M., Facey G.A., García H., García S., Scaiano J.C. (1996). Intrazeolite Photochemistry. 16. Fluorescence of Methylviologen Adsorbed within Medium- and Large-Pore Zeolites. J. Phys. Chem..

[28] Peon J., Tan X., Hoerner J.D., Xia C., Luk Y.F., Kohler B. (2001). Excited State Dynamics of Methyl Viologen. Ultrafast Photoreduction in Methanol and Fluorescence in Acetonitrile. J. Phys. Chem. A.

[29] Clennan E.L. (2004). Viologen embedded zeolites. Coord. Chem. Rev..

[30] Freitag M., Gundlach L., Piotrowiak P., Galoppini E. (2012). Fluorescence Enhancement of Di-p-tolyl Viologen by Complexation in Cucurbit uril. J. Am. Chem. Soc..

[31] Villemure G., Detellier C., Szabo A.G. (1986). Fluorescence of clay-intercalated methylviologen. J. Am. Chem. Soc..

[32] Alvaro M., García H., García S., Márquez F., Scaiano J.C. (1997). Intrazeolite Photochemistry. 17. Zeolites as Electron Donors:  Photolysis of Methylviologen Incorporated within Zeolites. J. Phys. Chem. B.

[33] Bâldea I., Köppel H., Wenzel W. (2013). (4,4′)-Bipyridine in vacuo and in solvents: A quantum chemical study of a prototypical floppy molecule from a molecular transport perspective. Phys. Chem. Chem. Phys..

[34] Reichardt C., Welton T. (2011). Solvents and Solvent Effects in Organic Chemistry.

[35] Papadakis R., Deligkiozi I., Tsolomitis A. (2010). Spectroscopic investigation of the solvatochromic behavior of a new synthesized non symmetric viologen dye: Study of the solvent-solute interactions. Anal. Bioanal. Chem..

[36] Papadakis R., Deligkiozi I., Tsolomitis A. (2012). Synthesis and characterization of a group of new medium responsive non-symmetric viologens. Chromotropism and structural effects. Dyes Pigment..

[37] Gutmann V. (1995). Lecture Notes on Solution Chemistry.

[38] Kamlet M.J., Abboud J.L.M., Abraham M.H., Taft R.W. (1983). Linear solvation energy relationships. 23. A comprehensive collection of the solvatochromic parameters, Pi.*, Alpha., and Beta., and some methods for simplifying the generalized solvatochromic equation. J. Org. Chem..

[39] Shi W., Xing F., Bai Y.-L., Hu M., Zhao Y., Li M.-X., Zhu S. (2015). High Sensitivity Viologen for a Facile and Versatile Sensor of Base and Solvent Polarity in Solution and Solid State in Air Atmosphere. ACS Appl. Mater. Inter..

[40] Hu M., Xing F., Zhao Y., Bai Y.-L., Li M.-X., Zhu S. (2017). Phenolacetyl Viologen as Multifunctional Chromic Material for Fast and Reversible Sensor of Solvents, Base, Temperature, Metal Ions, NH_3_ Vapor, and Grind in Solution and Solid State. ACS Omega.

[41] Reichardt C. (1994). Solvatochromic Dyes as Solvent Polarity Indicators. Chem. Rev..

[42] Gaina C., Gaina V., Airinei A., Avram E. (2004). Polyimides containing 4,4′-bipyridinium units. Appl. Polym. Sci..

[43] Wang Z., Tsarevsky N.V. (2016). Well-defined polymers containing a single mid-chain viologen group: Synthesis, environment-sensitive fluorescence, and redox activity. Polym. Chem..

[44] Sui Q., Ren X.-T., Dai Y.-X., Wang K., Li W.-T., Gong T., Fang J.-J., Zou B., Gao E.-Q., Wang L. (2017). Piezochromism and hydrochromism through electron transfer: New stories for viologen. Mater. Chem. Sci..

[45] Papadakis R., Deligkiozi I. (2019). Solvent Effects in Supramolecular Systems [Online First].

[46] Pramanik B., Mondal J.H., Singha N., Ahmed S., Mohanty J., Das D. (2017). A Viologen–Perylenediimide Conjugate as an Efficient Base Sensor with Solvatochromic Property. Chem. Phys. Chem..

[47] Gong T., Yang X., Fang J.-J., Sui Q., Xi F.-G., Gao E.-Q. (2017). Distinct Chromic and Magnetic Properties of Metal–Organic Frameworks with a Redox Ligand. ACS Appl. Mater. Interfaces.

[48] Li S.-L., Han M., Zhang Y., Li G.-P., Li M., He G., Zhang X.-M. (2019). X-ray and UV Dual Photochromism, Thermochromism, Electrochromism, and Amine-Selective Chemochromism in an Anderson-like Zn7Cluster-Based 7-Fold Interpenetrated Framework. J. Am. Chem. Soc..

[49] Yao Q.-X., Ju Z.-F., Jin X.-H., Zhang J. (2009). Novel Polythreaded Coordination Polymer: From an armed-Polyrotaxane Sheet to a 3D Polypseudorotaxane Array, Photo- and Thermochromic Behaviors. Inorg. Chem..

[50] Blandamer M.J., Burgess J., Haines R.I. (1976). Kinetic and equilibrium properties of pentacyano(3,5-dimethylpyridine)-iron(II) and related anions in mixed aqueous solvents. J. Chem. Soc. Dalton Trans..

[51] Toma H.E., Takasugi M.S. (1983). Spectroscopic studies of preferential and asymmetric solvation in substituted cyanoiron(II) complexes. J. Solution Chem..

[52] Pinheiro C., Lima J.C., Parola A.J. (2006). Using hydrogen bonding-specific interactions to detect water in aprotic solvents at concentrations below 50 ppm. Sens. Actuators B.

[53] Papadakis R., Tsolomitis A. (2009). Study of the correlations of the MLCT Vis absorption maxima of 4-pentacyanoferrate-40′-arylsubstituted bispyridinium complexes with the Hammett substituent parameters and the solvent polarity parameters ETN and AN. J. Phys. Org. Chem..

[54] Papadakis R., Tsolomitis A. (2011). Solvatochromism and preferential solvation of 4-pentacyanoferrate 4′-aryl substituted bipyridinium complexes in binary mixtures of hydroxylic and non-hydroxylic solvents. J. Solution Chem..

[55] Coe B.J., Harries J.L., Helliwell M., Jones L.A., Asselberghs I., Clays K., Brunschwig B.S., Harris J.A., Garín J., Orduna J. (2006). Pentacyanoiron(II) as an electron donor group for nonlinear optics: Medium-responsive properties and comparisons with related pentaammineruthenium(II) complexes. J. Am. Chem. Soc..

[B56-molecules-25-00001] 56. The term ”solvatochromic intensity” is used here by the author to express the solvatochromic response (CT wavelength, wavenumber, or energy change) induced by a certain input (change in solvent polarity expressed by a polarity parameter like Reichardt’s polarity scale *E**_T_*(30) [34]).

[57] Papadakis R. (2016). Preferential solvation of a highly medium responsive pentacyanoferrate(II) complex in binary solvent mixtures: Understanding the role of dielectric enrichment and the specificity of solute–solvent interactions. J. Phys. Chem. B..

[58] Papadakis R. (2014). The solvatochromic behavior and degree of ionicity of a synthesized pentacyano (N-substituted-4, 4′-bipyridinium) ferrate (II) complex in different media. Tuning the solvatochromic intensity in aqueous glucose solutions. Chem. Phys..

[59] Deligkiozi I., Voyiatzis E., Tsolomitis A., Papadakis R. (2015). Synthesis and characterization of new azobenzenecontaining bis pentacyanoferrate(II) stoppered push-pull [2]rotaxanes, with alpha- and beta-cyclodextrin. Towards highly medium responsive dyes. Dyes Pigment..

[60] Papadakis R., Deligkiozi I., Nowak K.E. (2019). Study of the preferential solvation effects in binary solvent mixtures with the use of intensely solvatochromic azobenzene involving [2] rotaxane solutes. J. Mol. Liq..

[61] Deligkiozi I., Papadakis R., Tsolomitis A. (2012). Synthesis, characterisation and photoswitchability of a new [2]rotaxane of α-cyclodextrin with a diazobenzene containing π-conjugated molecular dumbbell. Supramol. Chem..

[62] Baer A.J., Macartney D.H. (2000). α- and β-cyclodextrin rotaxanes of μ-Bis(4-pyridyl)bis[pentacyanoferrate(II)] Complexes. Inorg. Chem..

[63] Deligkiozi I., Papadakis R., Tsolomitis A. (2013). Photoconductive properties of a p-conjugated a-cyclodextrin containing [2]rotaxane and its corresponding molecular dumbbell. Phys. Chem. Chem. Phys..

[64] Papadakis R., Deligkiozi I., Li H. (2018). Photoconductive Interlocked Molecules and Macromolecules. Photodetectors.

[65] Yang X.-D., Zhu R., Yin J.-P., Sun L., Guo R.-Y., Zhan J. (2018). Bipyridinium-Bearing Multi-stimuli Responsive Chromic Material with High Stability. Cryst. Growth Des..

[66] Coe B.J., Fielden J., Foxon S.P., Helliwell M., Asselberghs I., Clays K., De Mey K., Brunschwig B.S. (2010). Syntheses and Properties of Two-Dimensional, Dicationic Nonlinear Optical Chromophores Based on Pyrazinyl Cores. J. Org. Chem..

[67] Coe B.J., Pilkington R.A. (2014). Theoretical Studies on Two-Dimensional Nonlinear Optical Chromophores with Pyrazinyl Cores and Organic or Ruthenium(II) Ammine Electron Donors. J. Phys. Chem. A.

[68] Sun X., James T.D. (2015). Glucose Sensing in Supramolecular Chemistry. Chem. Rev..

[69] De Borba E.B., Amaral C.L.C., Politi M.J., Villalobos R., Baptista M.S. (2000). Photophysical and Photochemical Properties of Pyranine/Methyl Viologen Complexes in Solution and in Supramolecular Aggregates:  A Switchable Complex. Langmuir.

[70] Brooks W.L.A., Sumerlin B.S. (2016). Synthesis and Applications of Boronic Acid-Containing Polymers: From Materials to Medicine. Chem. Rev..

[71] Hall D.G. (2011). Boronic Acids: Preparation and Applications in Organic Synthesis. Medicine and Materials.

[72] Gamsey S., Miller A., Olmstead M.M., Beavers M., Hirayama L.C., Pradhan S., Wessling R.A., Singaram B. (2007). Boronic Acid-Based Bipyridinium Salts as Tunable Receptors for Monosaccharides and α-Hydroxycarboxylates. J. Am. Chem. Soc..

[73] Sharrett Z., Gamsey S., Levine P., Cunningham-Bryant D., Vilozny B., Schiller A., Wessling R.A., Singaram B. (2008). Boronic acid-appended bis-viologens as a new family of viologen quenchers for glucose sensing. Tetrahedron Lett..

[74] Sharrett Z., Gamsey S., Fat J., Cunningham-Bryant D., Wessling R.A., Singaram B. (2007). The effect of boronic acid acidity on performance of viologen-based boronic acids in a two-component optical glucose-sensing system. Tetrahedron Lett..

[75] Cordes D.B., Gamsey S., Sharrett Z., Miller A., Thoniyot P., Wessling R.A., Singaram B. (2005). The Interact ion of Boronic Acid-Substituted Viologens with Pyranine: The Effects of Quencher Charge on Fluorescence Quenching and Glucose Response. Langmuir.

[76] Gamsey S., Suri J.T., Wessling R.A., Singaram B. (2006). Continuous Glucose Detection Using Boronic Acid-Substituted Viologens in Fluorescent Hydrogels: Linker Effects and Extension to Fiber Optics. Langmuir.

[77] Singaram B., Wessling R.A. (2003). Polyhydroxyl-substituted organic molecule sensing optical in vivo method utilizing a boronic acid adduct and the device thereof. US Patent.

[78] Foster R. (1969). Organic Charge-Transfer Complexes.

[79] Gutmann F., Johnson C., Keyzer H., Molnar J. (1997). Charge Transfer Complexes in Biological Systems.

[80] Saielli G. (2008). Ion-Pairing of Octyl Viologen Diiodide in Low-Polar Solvents: An Experimental and Computational Study. J. Phys. Chem. A.

[81] Nakahara A., Wang J.H. (1963). Charge transfer complexes of methylviologen. J. Phys. Chem..

[82] Kosower E.M., Cotter J.L. (1964). Stable Free Radicals. II. The Reduction of 1-Methyl-4-cyanopyridinium Ion to Methylviologen Cation Radical. J. Am. Chem. Soc..

[83] Curtis J.C., Sullivan B.P., Meyer T.J. (1980). Calculation of electron-transfer rate constants from the properties of charge-transfer absorption bands. The PQ^2+^, Fe(CN)_6_^4-^ system. Inorg. Chem..

[84] Watanabe T., Honda K. (1982). Measurement of the extinction coefficient of the methyl viologen cation radical and the efficiency of its formation by semiconductor photocatalysis. J. Phys. Chem..

[85] Bockman T.M., Kochi J.K. (1990). Isolation and oxidation-reduction of methylviologen cation radicals. Novel disproportionation in charge-transfer salts by X-ray crystallography. J. Org. Chem..

[86] Hammack W.S., Drickamer H.G., Hendrickson D.N. (1988). Effect of pressure on the charge-transfer band of the [Fe(CN)_6_]^4−^·dimethyl viologen ion pair. Chem. Phys. Lett..

[87] Monk P.M.S., Hodgkinson N.M., Partridge R.D. (1999). The colours of charge-transfer complexes of methyl viologen: Effects of donor, ionic strength and solvent. Dyes Pigment..

[88] Papadakis R., Deligkiozi I., Giorgi M., Faure B., Tsolomitis A. (2016). Supramolecular complexes involving nonsymmetric viologen cations and hexacyanoferrate (II) anions. A spectroscopic, crystallographic and computational study. RSC Adv..

[89] Tanaka R., Matsushi N. (2017). A charge-transfer salt composed of methyl viologen and hexacyanidoferrate(II). Acta Cryst. C.

[90] Antipin M.Y., Ilyukhin A.B., Kotov V.Y. (2001). Ice-like (H2O)12 and (H2O)14 clusters in the crystal structures of alkali metal–ethyl viologen hexacyanometallates. Mendeleev Commun..

[91] Tanaka R., Okazawa A., Konaka H., Sasaki A., Kojima N., Matsushita N. (2018). Unique Hydration/Dehydration-Induced Vapochromic Behavior of a Charge-Transfer Salt Comprising Viologen and Hexacyanidoferrate (II). Inorg. Chem..

[92] Kinuta T., Sato T., Tajima N., Kuroda R., Matsubara Y., Imai Y. (2010). Solid-state thermochromism observed in charge-transfer complex composed of binaphthol and viologen. J. Mol. Struct..

[93] Saielli G. (2019). Special Issue Editorial: Ionic Liquid Crystals. Crystals.

[94] Gabriel S., Weiner J. (1888). Ueber einige Abkömmlinge des Propylamins. Ber. Dtsch. Chem. Ges..

[95] Reinitzer F. (1888). Beiträge zur Kenntniss des Cholesterins. Monatsh. für Chem. (Wien).

[96] Cîrcu V., Handy S. (2019). Ionic Liquid C rystals Based on Pyridinium Salts, Progress and Developments in Ionic Liquids. Progress and Developments in Ionic Liquids.

[97] Saielli G. (2019). Ionic Liquid Crystals. Crystals 2019.

[98] Tabushi I., Yamamura K., Kominami K. (1986). Electric stimulus-response behavior of liquid-crystalline viologen. J. Am. Chem. Soc..

[99] Asaftei S., Ciobanu M., Lepadatu A.M., Song E., Beginn U. (2012). Thermotropic ionic liquid crystals by molecular assembly and ion pairing of 4,4′-bipyridinium derivatives and tris(dodecyloxy)benzenesulfonates in a non-polar solvent. J. Mater. Chem..

[100] Bonchio M., Carraro M., Casella G., Causin V., Rastrelli F., Saielli G. (2012). Thermal behaviour and electrochemical properties of bis(trifluoromethanesulfonyl)amide and dodecatungstosilicate viologen dimers. Phys. Chem. Chem. Phys..

[101] Gunaratne H.Q.N., Nockemann P., Olejarz S., Reid S.M., Seddon K.R., Srinivasan G. (2013). Ionic Liquids with Solvatochromatic and Charge-Transfer Functionalities Incorporating the Viologen Moiety. Austr. J. Chem..

[102] Wang R.-T., Lee G.-H., Lai C.K. (2018). Anion-induced ionic liquid crystals of Diphenylviologens. J. Mater. Chem. C.

[103] Kobayashi T., Ichikawa T. (2017). Design of Viologen-Based Liquid Crystals Exhibiting Bicontinuous Cubic Phases and Their Redox-Active Behavior. Materials.

[104] Mortimer R.J., Rosseinsky D.R., Monk P.M.S. (2015). Electrochromic Materials and Devices.

